# Combining Landscape Genomics and Ecological Modelling to Investigate Local Adaptation of Indigenous Ugandan Cattle to East Coast Fever

**DOI:** 10.3389/fgene.2018.00385

**Published:** 2018-10-03

**Authors:** Elia Vajana, Mario Barbato, Licia Colli, Marco Milanesi, Estelle Rochat, Enrico Fabrizi, Christopher Mukasa, Marcello Del Corvo, Charles Masembe, Vincent B. Muwanika, Fredrick Kabi, Tad Stewart Sonstegard, Heather Jay Huson, Riccardo Negrini, The NextGen Consortium, Stéphane Joost, Paolo Ajmone-Marsan

**Affiliations:** ^1^Department of Animal Science, Food and Nutrition (DIANA), Biodiversity and Ancient DNA Research Centre (BioDNA), and Proteomics and Nutrigenomics Research Centre (PRONUTRIGEN), Università Cattolica del Sacro Cuore, Piacenza, Italy; ^2^Laboratory of Geographic Information Systems (LASIG), School of Architecture, Civil and Environmental Engineering (ENAC), École Polytechnique Fédérale de Lausanne (EPFL), Lausanne, Switzerland; ^3^Department of Support, Production and Animal Health, School of Veterinary Medicine, São Paulo State University, Araçatuba, Brazil; ^4^International Atomic Energy Agency (IAEA), Collaborating Centre on Animal Genomics and Bioinformatics, Araçatuba, Brazil; ^5^Department of Economics and Social Sciences, Università Cattolica del Sacro Cuore, Piacenza, Italy; ^6^National Animal Genetic Resource Centre and Data Bank, Entebbe, Uganda; ^7^Department of Zoology, Entomology and Fisheries, Makerere University, Kampala, Uganda; ^8^Department of Environmental Management, Makerere University, Kampala, Uganda; ^9^National Livestock Resources Research Institute (NaLIRRI), National Agricultural Research Organisation, Tororo, Uganda; ^10^Recombinetics, Inc., St. Paul, MN, United States; ^11^Department of Animal Science, Cornell University, Ithaca, NY, United States; ^12^Associazione Italiana Allevatori (AIA), Rome, Italy; ^13^http://nextgen.epfl.ch/consortium

**Keywords:** local adaptation, landscape genomics, species distribution modelling, indigenous cattle, East Coast fever

## Abstract

East Coast fever (ECF) is a fatal sickness affecting cattle populations of eastern, central, and southern Africa. The disease is transmitted by the tick *Rhipicephalus appendiculatus*, and caused by the protozoan *Theileria parva parva*, which invades host lymphocytes and promotes their clonal expansion. Importantly, indigenous cattle show tolerance to infection in ECF-endemically stable areas. Here, the putative genetic bases underlying ECF-tolerance were investigated using molecular data and epidemiological information from 823 indigenous cattle from Uganda. Vector distribution and host infection risk were estimated over the study area and subsequently tested as triggers of local adaptation by means of landscape genomics analysis. We identified 41 and seven candidate adaptive loci for tick resistance and infection tolerance, respectively. Among the genes associated with the candidate adaptive loci are *PRKG1* and *SLA*2. *PRKG1* was already described as associated with tick resistance in indigenous South African cattle, due to its role into inflammatory response. *SLA*2 is part of the regulatory pathways involved into lymphocytes' proliferation. Additionally, local ancestry analysis suggested the zebuine origin of the genomic region candidate for tick resistance.

## Introduction

East Coast fever (ECF) is an endemic vector-borne disease affecting the species *Bos taurus* in eastern and central Africa. ECF etiological agent is the emo-parasite protozoan *Theileria parva* Theiler, 1904, vectored by the hard-bodied tick vector *Rhipicephalus appendiculatus* Neumann, 1901. The disease is reported to cause high morbidity and mortality in susceptible indigenous populations coming from outside endemic areas and among exotic breeds, thus undermining the livestock sector development in the affected countries (Norval et al., 1992; Olwoch et al., 2008; Muhanguzi et al., 2014).

Cape buffalo (*Syncerus caffer* Sparrman, 1779) is *T. parva* native host, being its wild and asymptomatic reservoir (Oura et al., 2011). The primordial contact between buffalo-derived *T. parva* and domestic bovines probably occurred ~4500 years before present (YBP), following *B. taurus* migration into *T. parva* areal (Epstein, 1971). However, it is hard to define if the host jump affected taurine- or indicine-like *B. taurus* first, since no consensus can easily be reached to define who among the subspecies *B. t. taurus* and *B. t. indicus* was present in East Africa at that time (Freeman, 2006; Hiendleder et al., 2008; Decker et al., 2014; Magee et al., 2014; Mwai et al., 2015). In particular, African taurine migration might have occurred sometime between ~8,000 and 1,500 YBP (Magee et al., 2014; Mwai et al., 2015), and the most ancient zebuine colonization wave is estimated to have occurred between ~4,000 and 2,000 YBP from the Asian continent, as suggested by the first certain archaeological record dated 1,750 YBP (Freeman, 2006). Once *T. parva* spread to domestic populations, coevolution between the parasite and the new hosts likely led to the divergence between buffalo- (*T. p. lawracei*) and cattle-specific (*T. p. parva*) parasite strains (Hayashida et al., 2013; Sivakumar et al., 2014), and to the appearance of indigenous herds able to survive and recover from infection (Kabi et al., 2014; Bahbahani and Hanotte, 2015).

Most likely, such populations appeared (and still inhabit) areas where environmental conditions guaranteed the constant coexistence between vector, parasite, and domestic host. Such a combination, together with the evolution of some sort of resistance and/or tolerance (i.e., the capacity of reducing parasite burden or attenuating the symptoms caused by a given parasite burden, respectively), plausibly prompted the establishment of an epidemiological state referred to as endemic stability, a condition where hosts become parasite reservoirs while showing negligible clinical symptoms (Kivaria et al., 2004; Råberg et al., 2007; Gachohi et al., 2012; Laisser et al., 2017). However, to our knowledge no clear indication has been provided for a genetic control prompting survival and recovery from ECF (Bahbahani and Hanotte, 2015), although previous research identified the role of host genetics on both tolerance and resistance strategies in animals (Råberg et al., 2007), as in the case of *B. taurus* resistance to tropical theileriosis (Glass and Jensen, 2007; Chaussepied et al., 2010) and tick burden (Kim et al., 2017). Furthermore, the identification of adaptive variation responsible for survival to ECF would undoubtedly represent a sensible step forward toward sustainability and productivity of local agroecosystems.

Here, we explicitly test the hypothesis that indigenous host populations living in ECF-endemically stable areas are locally adapted to ECF burden, and investigate for selection signatures involved with ECF-tolerance/resistance. In particular, local adaptation is known to be promoted by spatially varying (i.e., divergent) natural selection (Kawecki and Ebert, 2004), leading a population at its native site to present higher fitness “than any other population introduced to that site” (Savolainen et al., 2013). Such conditions appear to be met in East Africa, where *R. appendiculatus* distribution is reported to be wide but patchy (implying a spatially heterogeneous ECF burden on local host populations; Olwoch et al., 2003, 2008), *B. taurus* presence is long-standing and its distribution wide (Robinson et al., 2014), and the introduction of allochthonous *B. taurus* populations into ECF-endemic areas proved unsuccessful, with mortality rates ranging 40–100% (Rubaire-Akiiki et al., 2006; Olwoch et al., 2008; Gachohi et al., 2012).

By testing for significant associations between environmental and genetic features of individuals (or populations) at their sampling sites, landscape genomics aims to detect the environmental drivers of divergent selection triggering adaptive variation (Rellstab et al., 2015). Here, we relied on a landscape genomics approach to search for signatures of local adaptation in the genomes of indigenous *B. taurus* populations from Uganda, where the concomitant occurrence of endemically stable areas in the South-West and in the East of the country (Kivaria et al., 2004; Rubaire-Akiiki et al., 2006), spatially varying selection due to regional climatic differences (Kabi et al., 2014), and host populations connected by high rates of gene flow (Kawecki and Ebert, 2004; Stucki et al., 2017) is likely to have promoted local adaptation to the disease even over short time scales, i.e., from thousands of years to few decades (Stockwell et al., 2003; Crispo et al., 2010; Fraser et al., 2011).

Since endemic areas are currently inhabited by indigenous *B. t. indicus* and the *B. t. indicus x* African *B. t. taurus* crosses sanga and zenga (Hanotte et al., 2002; Mwai et al., 2015), two main hypotheses can be associated with the origin of local adaptation to ECF: (i) at first adaptation appeared in local African *B. t. taurus* populations and was then introgressed into zebu and derived sanga and zenga crossbreds; alternatively, (ii) it appeared in *B. t. indicus*, and then either evolved independently in zebuine populations of eastern Africa, or was imported from the Indian continent, where similar selective pressures are recorded (Singh et al., 1993; Boulter and Hall, 1999).

To search for ECF-specific signatures of selection, we first modelled *R. appendiculatus* potential distribution and *T. p. parva* infection risk in Uganda to define the spatially varying selective pressure over the host genomes, and then used this information to search for single-nucleotide polymorphisms (SNPs) potentially involved into local adaptation to ECF through genotype-environment association analysis. Subsequently, we annotated candidate genes, and studied the ancestral origin of the identified candidate genomic regions by means of local ancestry analysis to shed light on the possible evolutionary origins of local adaptation.

## Materials and methods

### Ecological modelling

*R. appendiculatus* occurrence probability (ψ_*R*_) and *T. p. parva* infection risk in cattle (γ) were modelled and used as environmental predictors into landscape genomics models. Geographical variability in both ψ_*R*_ and γ was assumed to describe the spatially heterogeneous selective pressure on cattle genomes. Further, *S. caffer* occurrence probability (ψ_*S*_) was estimated and used in combination with ψ_*R*_ to model γ, as the geographical proximity between Cape buffaloes and cattle herds constitutes a factor for explaining ECF incidence. The following three sections will describe data and methods used to estimate ψ_*R*_, ψ_*S*_, and γ.

#### Raster data

Bioclimatic variables (BIO) referring to the time span between 1960 and 1990 were collected from the WorldClim database (v.1.4. release3) (Hijmans et al., 2005) at a spatial resolution of 30 arc-seconds and in the un-projected latitude/longitude coordinate reference system (WGS84 datum). Altitude information was collected from the SRTM 90 m Digital Elevation Database (v.4.1) (Jarvis et al., 2008), which provides tiles covering Earth's land surface in the WGS84 datum, at 90 m resolution at the equator. Altitude was used to compute terrain slope through the function terrain implemented in the R package raster (Hijmans, 2016). The 10-year (2001–2010) averaged Normalized Difference Vegetation Index (NDVI) was derived for 72 ten-day annual periods from the “eMODIS products” (Supplementary Text 1) (US Geological Survey^1^), in the WGS84 datum, and at a resolution of 250 m at the equator. A raster file describing cattle density (number of animals/km^2^) was acquired from the Livestock Geo-Wiki database (Robinson et al., 2014), in the WGS84 datum, at a resolution of 1 km^2^ at the equator. A raster file describing each pixel distance from the nearest water source was obtained with the function distance within the R package raster. The “Land and Water Area” dataset from the Gridded Population of the World collection (GPV v.4) (CIESIN, 2016) was used to define water bodies in Uganda at a resolution of 30 arc-seconds with WGS84 datum.

All raster files were transposed into Africa Albers Equal Area Conic projection to guarantee a constant pixel size and meet the main assumption of the statistical technique used to model ψ_*R*_ and ψ_*S*_, i.e., that each pixel presents the same probability to be randomly sampled in order to detect the species occurrence (Merow and Silander, 2014). Raster files were standardised to the same resolution (~0.85 km^2^), origin, and extent. To avoid the inclusion of potentially misleading background locations while characterizing the occurrence probability of terrestrial species, inland water surfaces were masked prior to ψ_*R*_ and ψ_*S*_ estimation (Barve et al., 2011). Quantum GIS (v.2.16.2) (QGIS Development Team, 2016) and the R package raster were used for raster files manipulation.

#### Species distribution models

The R package Maxlike (Royle et al., 2012) was used to model ψ_*R*_ and ψ_*S*_ over Uganda. Maxlike is able to estimate species occurrence probability (ψ) from presence-only data, by maximizing the likelihood of occurrences under the logit-linear model (Merow and Silander, 2014):

$$\begin{array}{l} ln\left(\frac{\psi_{x}}{1-\psi_{x}}\right)=\beta_{0}+\beta z\left(x\right) \end{array}$$

where ψ_*x*_ denotes the species occurrence probability in the *x* pixel of the landscape, β_0_ the model intercept (i.e., the expected prevalence across the study area), β the vector of slope parameters, and *z(x)* the vector of environmental variables for *x*. Occurrence probability in *x* is derived from the inverse logit:

$$\begin{array}{l} \psi_{x}=\frac{e^{\beta_{0}+\beta z\left(x\right)}}{1+e^{\beta_{0}+\beta z\left(x\right)}} \end{array}$$

**Figure 1:**
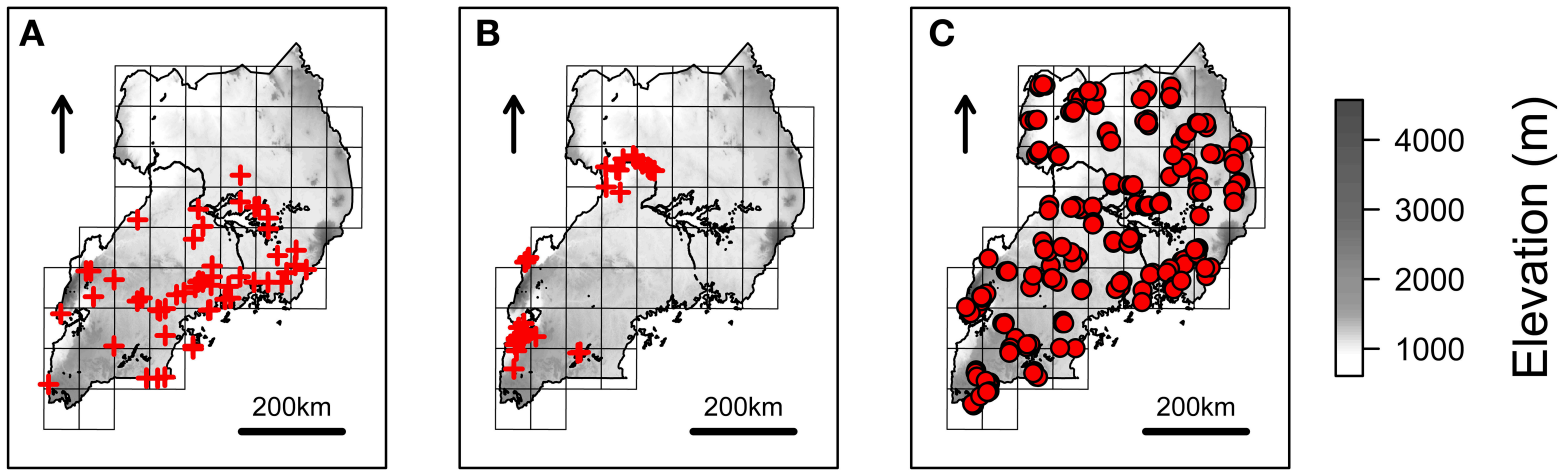
Species occurrences and NextGen sampling scheme. Red crosses represents the spatial records used to estimate *Rhipicephalus appendiculatus* **(A)** and *Syncerus caffer* **(B)** distributions over Uganda, as derived from Cumming (1999b) and GBIF (2012), respectively. Farms where cattle have been sampled to be genotyped and tested for *Theileria parva parva* infection are represented with red circles **(C)**. The grid scheme used to sample farms during the NextGen project is shown on the background of each map (see main text), together with elevation.

Fifty-one and 61 spatial records of *R. appendiculatus* and *S. caffer* (Figures 1A,B) were obtained from a tick occurrence database previously collected (Cumming, 1999b), and the Global Biodiversity Information Facility (GBIF, 2012), respectively.

The most relevant environmental variables affecting tick and Cape buffalo distributions were identified from the literature. Specifically, the BIO variables representing temperature/precipitation interaction in the most extreme periods of the year were used to model *R. appendiculatus* occurrence (Table 1 and Supplementary Figure 1) (Cumming, 1999a, 2002), while altitude, terrain slope, NDVI, distance to water sources (Wd), and annual precipitation (BIO_12_) were used to model the Cape buffalo distribution (Pettorelli et al., 2011; Matawa et al., 2012; Naidoo et al., 2012). A Maxlike regression analysis was applied to individuate the NDVI values best predicting the available *S. caffer* occurrences, and the period April 6–15 was retained for subsequent analyses (Supplementary Figure 2). No variable depicting the top-down regulatory effect of predators on buffalo populations was considered, as bottom-up ecological mechanisms (like quantity and quality of food resources) are argued to play the main role in determining large herbivores distribution (Winnie et al., 2008).

**Table 1:** Predictors used to model *Rhipicephalus appendiculatus* distribution.

**Bioclim variable**	**Definition**
BIO_8_	Mean temperature ^a^ of the wettest 3 months (quarter) of the year
BIO_9_	Mean temperature of the driest quarter
BIO_10_	Mean temperature of the warmest quarter
BIO_11_	Mean temperature of the coldest quarter
BIO_16_	Precipitation^b^ of the wettest quarter
BIO_17_	Precipitation of the driest quarter
BIO_18_	Precipitation of the warmest quarter
BIO_19_	Precipitation of the coldest quarter

a *Temperature was transformed from dC° to C° prior analyses*.

b *Precipitation is expressed in millimetres*.

Collinearity was checked prior to analyses by computing pairwise absolute correlations (|*r*|) between variables, which were considered collinear when |*r*| exceeded the suggested threshold of 0.7 (Dormann et al., 2013). High collinearity was found among BIO variables, which were then subjected to principal components analysis (PCA) to obtain orthogonal predictors for ψ_*R*_.

Obtained components were tested into univariate and multivariate *R. appendiculatus* distribution models. Particularly, components explaining up to 95% of the original variance (Jolliffe, 2002) were individuated and tested with different combinations into multivariate models, leading to a total of 12 candidate *R. appendiculatus* distribution models. Conversely, all the combinations of environmental variables were tested into univariate up to penta-variate Cape buffalo distribution models, resulting in a total of 31 candidate models for predicting *S. caffer* potential distribution.

In both cases, Bayesian Information Criterion (BIC) was used to select the best models (Aho et al., 2014). Bring's standardization (Bring, 1994; Cade, 2015) was applied on predictors before parameters' estimate, and the delta method was implemented to compute the 95% confidence intervals around the fitted ψ_*Rx*_ and ψ_*Sx*_.

#### Infection risk model

In the context of the European Project NextGen^2^, 587 blood samples from Ugandan indigenous *B. taurus* were tested for the presence/absence of *T. p. parva* p104 antigen DNA sequence (Kabi et al., 2014). Samples were collected and georeferenced in correspondence of 203 farms distributed over a grid of 51 cells covering the whole Uganda, with an average of 12 (±4 s.d.) animals/cell, and three (±1 s.d.) animals/farm (Figure 1C).

ECF epidemiology is complex and determined by both biotic and abiotic factors (Norval et al., 1992). Particularly, *R. appendiculatus* occurrence (ψ_*R*_) (Magona et al., 2008, 2011; Gachohi et al., 2011; Muhanguzi et al., 2014), cattle density (Cd) (Billiouw et al., 2002; Olwoch et al., 2008), potential proximity with *S. caffer* (ψ_*S*_) and the maximal temperature in the warmest month of the year (BIO_5_) were considered to predict γ. BIO_5_ was used to account for the possible limiting effect of high temperatures on the parasite development into the tick (Young and Leitch, 1981). Predictors' values were obtained at the geographical position of each animal (i.e., at farm locations), checked for the presence of collinearity (as done for the species distribution models) and outliers (Supplementary Figure 3), and subsequently standardized following Bring's procedure prior to parameters' estimation.

Infection risk for any *i-th* animal was modelled using a binary mixed-effects logistic regression, where ψ_*R*_, BIO_5_, Cd, and ψ_*S*_ were specified as fixed effects, and random intercepts were estimated for each farm to account for the possible influence of local environmental conditions and management practices (e.g., differential use of acaricides), as well as unmeasured biological features (e.g., breed- or individual-specific response to tick burden) (Gachohi et al., 2012). Since geographical position of samples was recorded at the farm-level, all the animals coming from a given farm were characterized by equal environmental values. Thus, the model can be written as:

$$\begin{array}{r} ln\left(\frac{\gamma_{ij}}{1-\gamma_{ij}}\right)=\left({\;\beta }_{0}+b_{0j}\right)+\beta z\left(j\right)\\ b_{0j}\sim \text{N}\left(0,\sigma_{b_{0}}^{2}\right) \end{array}$$

where γ_*ij*_ represents *T. p. parva* infection risk for the *i-th* animal in the *j-th* farm, β_0_ is the population intercept (Zuur et al., 2009), β_0_+*b*_0*j*_ is the *j-th* farm random intercept, β the vector of slope parameters, and *z(j)* the vector containing the predictors' values as derived from the pixel where the *j-th* farm is located, equal for all the animals in *j*. In this way, animals in *j* are expected with the same predicted γ, so that infection risk in the *j-th* farm can be calculated using the population model from the previous equation:

$$\begin{array}{l} \gamma_{j}=\frac{e^{\beta_{0}+\beta z\left(j\right)}}{1+e^{\beta_{0}+\beta z\left(j\right)}} \end{array}$$

Estimates of the parameters were obtained through the Maximum Likelihood criterion using the glmer function included in the R package lme4 (Bates et al., 2015).

### Landscape genomics

#### Molecular datasets

The NextGen project genotyped 813 georeferenced indigenous *B. taurus* from Uganda using the medium-density BovineSNP50 BeadChip (54,596 SNPs; Illumina Inc., San Diego, CA, USA). Landscape genomics analyses were carried out on this set of animals, which will be referred to as the “landscape genomics dataset” (LGD). Samples were collected according to the spatial scheme represented in Figure 1C, and encompassed 503 of the individuals tested for *T. p. parva* infection. Quality control (QC) procedures were carried out with the software Plink v.1.7 (Purcell et al., 2007). LGD was limited to autosomal chromosomes and pruned for minor allele frequency (MAF) <0.01, genotype call rates <0.95, and individual call rate <0.9. Pairwise genome-wide identity-by-descent (IBD) values were estimated, and one individual per pair showing IBD>0.5 was excluded from analyses to reduce the risk of spurious associations due to unreported kinship (Turner et al., 2011). To avoid excluding too many individuals from nearby areas, spatial positions of the highlighted pairs were considered prior to removal.

Population genetic structure of Ugandan cattle was studied on the landscape genomics dataset merged with molecular data from other European taurine, African taurine, zebuine, and sanga populations retrieved from various sources and for different geographical areas worldwide (Supplementary Table 1). This extended dataset will be referred to as the “population structure dataset” (PSD). Plink was used to prune PSD for linkage disequilibrium (LD) >0.1 with sliding windows of 50 SNPs and step size of 10 SNPs (option –indep-pairwise 50 10 0.1), and to filter for the QC thresholds previously reported.

#### Population structure analysis

PSD was analysed with admixture v.1.3.0 (Alexander et al., 2009) for a dual purpose. Firstly, to provide genotype-environment association tests with population structure predictors in order to reduce the risk of false positive detections (Schoville et al., 2012; Rellstab et al., 2015). To this aim, we decided to use membership coefficients for the four-cluster solution (*K*=4), as this was reported to be the best partition based on the admixture cross-validation index for the same set of Ugandan individuals undergoing landscape genomics in the present study (Stucki et al., 2017). Due to strong collinearity (|*r*|>0.7) (Dormann et al., 2013) among the membership coefficients of two ancestral components, a PCA was performed trough the R function prcomp to obtain synthetic and orthogonal population structure predictors. Secondly, to identify the main gene pools present in Uganda in the context of a worldwide-extended dataset, and therefore guide selection of proper reference populations for local ancestry analysis.

#### Genotype-environment associations

We used the software samβada v.0.5.3 (Joost et al., 2007; Stucki et al., 2017) to test for associations between *B. taurus* genotypes and ψ_*R*_ and γ at sampling locations. Given diploid species and biallelic markers, samβada runs three models per locus, one for each possible genotype. Each model estimates the probability π_*i*_ for the *i-th* individual to carry a given genotype, as a function of the considered environmental and population structure variables:

$$\begin{array}{l} \ln\left(\frac{\pi_{i}}{1-\pi_{i}}\right)=\beta_{0}+\beta z\left(i\right) \end{array}$$

and thus:

$$\begin{array}{l} \pi_{i}=\frac{e^{\beta_{0}+\beta z\left(i\right)}}{1+e^{\beta_{0}+\beta z\left(i\right)}} \end{array}$$

Genotype-environment association tests were carried out through a likelihood-ratio test comparing a null and an alternative model for each genotype (Stucki et al., 2017). Particularly, null models comprised the population structure predictors alone, while alternative ones included population structure predictors plus either ψ_*R*_ or γ. A genotype was considered significantly associated with ψ_*R*_ and/or γ if the resulting *p-value* associated with the likelihood-ratio test statistic (*D*) was lower than the nominal significance threshold of 0.05 after Benjamini-Hochberg (BH) correction for multiple testing (*H*_0_: *D*=0, α_BH_=0.05; Supplementary Texts 2–3). The R function p.adjust was used to perform *p-values* corrections, and predictors were centred prior to analysis to ease estimation of model parameters.

#### Gene annotation

Global linkage disequilibrium (LD) decay was estimated using snep v.1.11 (Barbato et al., 2015) to define LD extent around marker loci. A window of ±25 kbp (*r*^2^≈0.2) was then selected around those SNPs associated with ψ_*R*_ and/or γ to annotate genes in the Ensembl database release 87 (Aken et al., 2016). Annotated genes were investigated for known biological function according to the literature, and candidate genes identified based on their pertinence with ECF local adaptation.

### Local ancestry

#### Molecular dataset

Target population for local ancestry analysis comprised 102 indigenous Ugandan *B. taurus* individuals collected during the NextGen sampling campaign (two animals sampled per cell; Figure 1C), and genotyped with the BovineHD BeadChip (777,961 SNPs; Illumina Inc., San Diego, CA, USA). Reference populations (see Results section) were selected in coherence with the major Ugandan gene pools identified by the Admixture analysis (Supplementary Text 4). Target and reference populations were pooled in a “local ancestry dataset” (LAD). Only autosomal SNPs passing the same filtering parameters applied to LGD were retained for analysis.

#### PCAdmix analysis

Local ancestry investigation allows to assign the ancestral origin of a chromosomal region (window) given two or more reference populations, and have been used to infer the admixture history of closely related groups (Pasaniuc et al., 2009), identify signals of adaptive introgression (Barbato et al., 2017), and highlight target regions of recent selection (Tang et al., 2007). Here, PCAdmix v.1.0 (Brisbin et al., 2012) was used to infer local genomic ancestry of the Ugandan samples. Given the SNPs density present in LAD (i.e., one SNP every ~3.4 kbp, on average), we used 20 SNPs per window to obtain a window size comparable to the optimal one suggested in Brisbin et al. (2012).

#### Beta regression analysis

Genomic windows hosting SNPs in linkage with the candidate genes for local adaptation were identified and their ancestry proportions computed per sampling cell (Figure 1C). Average ψ_*R*_ and γ per cell values (hereafter ψ_*Rc*_ and γ_*c*_, respectively) were derived using the zonal.stats function included in the R package spatialEco (Evans, 2017). In order to test for significant associations between ancestry proportions and ψ_*Rc*_ and γ_*c*_, a beta regression analysis was performed using the R package betareg (Cribari-Neto and Zeileis, 2010), according to the model:

$$\begin{array}{r} \ln\left(\frac{\mu_{i}}{1-\mu_{i}}\right)=\beta_{0}+{\beta_{1}x}_{i}\\ a_{i}\sim \left(\mu_{i},\phi \right) \end{array}$$

where *a*_*i*_ is the ancestry proportion observed in cell *i*, which is assumed to derive from a beta distribution B(μ_*i*_, ϕ) with mean μ_*i*_=E(*a*_*i*_) and precision parameter ϕ, *x*_*i*_ is either average ψ_*R*_ or γ in cell *i*, β_0_ and β_1_ are intercept and regression coefficient, respectively. Expected ancestry proportion in *i* was calculated through the inverse logit:

$$\begin{array}{l} \mu_{i}=\frac{e^{\beta_{0}+{\beta_{1}x}_{i}}}{1+e^{\beta_{0}+{\beta_{1}x}_{i}}} \end{array}$$

Ancestry proportions were transformed prior to analysis (Smithson and Verkuilen, 2006), and the Maximum Likelihood criterion was used to estimate model parameters.

### Ethics statement

The NextGen sampling campaign was carried out during years 2011 and 2012, before Directive 2010/63/EU came into force (i.e., 1 January 2013). Thus, all experimental procedures were compliant with the former EU Directive 86/609/EEC, according to which no approval from dedicated animal welfare/ethics committee was needed for this study. The permission to carry out the study was obtained from the Uganda National Council for Science and Technology (UNCST) reference number NS 325 (Kabi et al., 2014). The permission to carry out the sampling at each farm was obtained directly from the owners.

## Results

### Ecological modelling

#### Species distribution models

**Figure 2:**
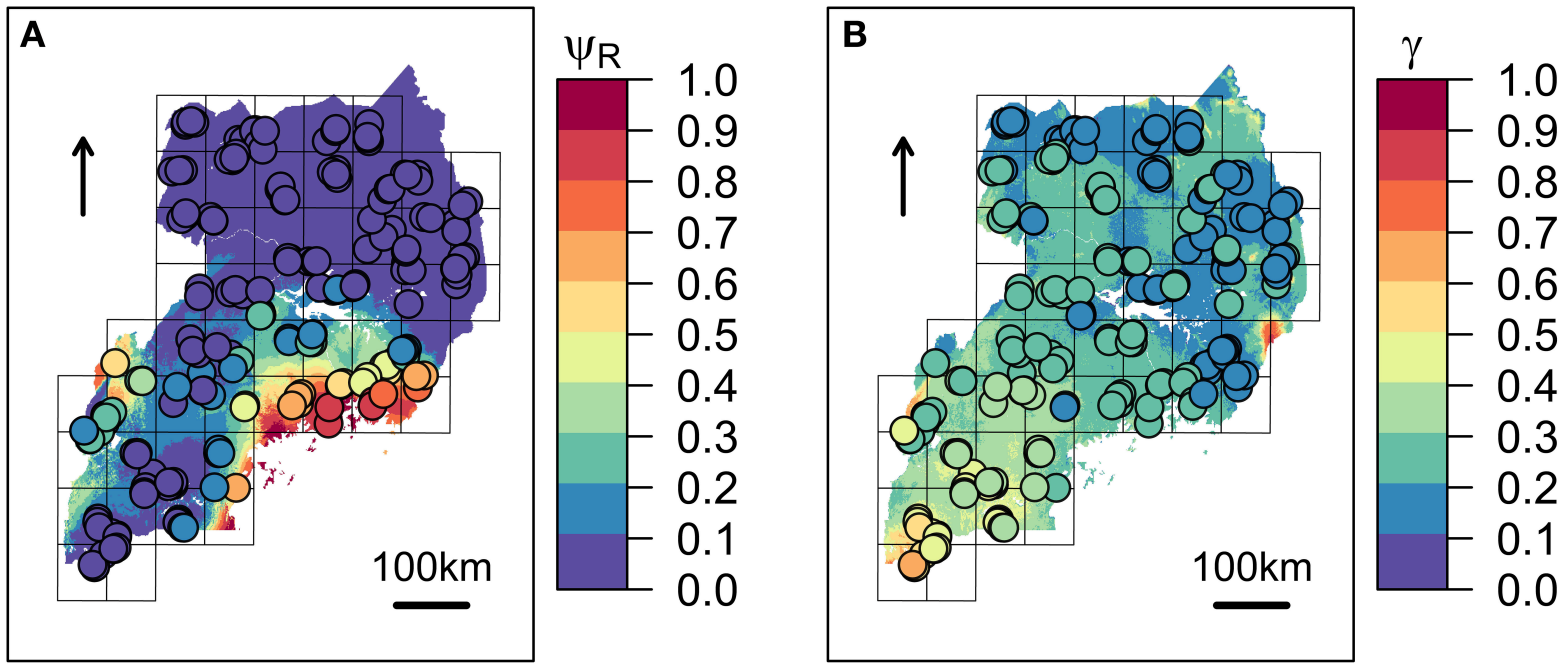
Predicted spatial distributions for ECF vector and infection risk in cattle. **(A)** *Rhipicephalus appendiculatus* occurrence probability (ψ_*R*_) as predicted by the selected distribution model. **(B)** Predicted *Theileria parva parva* infection risk (γ). Colour from blue to red tones corresponds to increasing values of ψ_*R*_ and γ. Sampled farms are represented with circles, and coloured according to ψ_*R*_ and γ values estimated at their geographical location.

The first three principal components (PC_1_, PC_2_, and PC_3_) accounted for more than 95% of the variance among the BIO predictors, and were subsequently tested into multivariate Maxlike models to estimate ψ_*R*_. Particularly, PC_1_ (61%) was mainly correlated with BIO variables linked to temperature (BIO_8_, BIO_9_, BIO_10_, and BIO_11_), PC_2_ (19%) with precipitation (BIO_16_, BIO_17_, BIO_18_, and BIO_19_), and PC3 (15%) with both temperature and precipitation (BIO_19_ and BIO_8_) (Table 1 and Supplementary Figure 5). The model employing PC_1_, PC_2_, and PC_3_ was selected based on the BIC metric (Supplementary Figure 6), with PC_1_, and PC_2_ showing a significant positive effect on the tick distribution, and PC_3_ a significant negative effect (Table 2) (*H*_0_: β_*i*_=0, α=0.05). The model predicts low habitat suitability in the regions North of the Lakes Kwania, Kyoga and Kojwere (0<ψ_*R*_<0.1), and favourable ecological conditions around Lake Victoria (0.4<ψ_*R*_<1) and South-West of Lake Albert (0.4<ψ_*R*_<0.8), these latter separated by a corridor of lower suitability (0<ψ_*R*_<0.3) (Figure 2A and Supplementary Figure 7).

**Table 2:** Maxlike results for *Rhipicephalus appendiculatus* distribution model.

**Coefficient**	**Estimate**	**SE**	***p-value***	**OR^a^**	**OR_low_^b^**	**OR_up_^c^**
β_0_	−2.905	0.561	2.24E−07^***^	0.055	0.018	0.164
PC_1_	0.796	0.303	8.56E−03^**^	2.217	1.224	4.014
PC_2_	0.822	0.37	2.62E−02^*^	2.275	1.102	4.698
PC_3_	−1.799	0.629	4.27E−03^**^	0.165	0.048	0.568

Point estimates (Estimate) of the standardized regression coefficients (Coefficient) are reported on the logit scale together with their standard errors (SE), p-values and the associated odds ratios (OR). A significant effect is reported with

*** *when the p-value (p) associated to a regression coefficient is ≤ 0.001*;

** when 0.001 < p < 0.01;

* *when 0.01 < p < 0.05*.

a *Odds ratios associated with regression coefficients express the expected change in the ratio ψ_R_/(1–ψ_R_), for a one standard deviation increase of the concerned predictor, holding all the other predictors fixed at a constant value*.

b *Odds ratio 95% confidence interval (CI), lower bound*.

c *Odds ratio 95% CI, upper bound*.

No excessive collinearity was recorded among the predictors for ψ_*S*_. The best model according to the BIC metric included: altitude, annual precipitation, average NDVI, and distance from the nearest water source (Table 3 and Supplementary Figure 8). The model predicts the highest habitat suitability (0.2<ψ_*S*_<0.8) in the near proximity of the water bodies (especially along the White Nile in the North-West, the south-eastern coasts of Lake Édouard, and the northern coasts of Lake George), and in small areas near the Katonga Game Reserve (Supplementary Figure 9).

**Table 3:** Maxlike results for *Syncerus caffer* distribution model.

**Coefficient**	**Estimate**	**SE**	***p-value***	**OR^a^**	**OR_low_^b^**	**OR_up_^c^**
β_0_	−9.130	0.790	6.46E−31^***^	0.000	0.000	0.001
Altitude	−1.095	0.293	1.90E−04^***^	0.335	0.188	0.594
BIO_12_	−0.800	0.180	9.03E−06^***^	0.449	0.316	0.639
NDVI	2.862	0.329	3.38E−18^***^	17.499	9.181	33.343
Wd	−1.996	0.434	4.23E−06^***^	0.136	0.058	0.318

Point estimates (Estimate) of the standardized regression coefficients (Coefficient) are reported on the logit scale together with their standard errors (SE), p-values and the associated odds ratios (OR). Significant regression coefficients are highlighted with

*** when their p-values (p) are ≤ 0.001; ^**^when 0.001 < p ≤ 0.01; ^*^when 0.01 < p ≤ 0.05.

a *Odds ratios associated with regression coefficients express the expected change in the ratio ψ_S_/(1–ψ_S_), for a one standard deviation increase of the concerned predictor, holding all the other predictors fixed at a constant value*.

b *Odds ratio 95% confidence interval (CI), lower bound*.

c *Odds ratio 95% CI, upper bound*.

#### Infection risk model

Following outliers inspection, ψ_*R*_, Cd, and ψ_*S*_ were transformed on the log_10_ scale to reduce the observed skewness in the distributions (Supplementary Figure 3). No excessive collinearity was observed among the model predictors (|*r*|<0.7). All the explanatory variables except for Cd showed a significant effect (*H*_0_: β_*i*_=0, α=0.05) on infection risk. Particularly, BIO_5_ and ψ_*R*_ showed a negative association with γ, while ψ_*S*_ resulted positively associated (Table 4). Overall, northern regions of Uganda present a low probability of infection (0.1<γ<0.3). A similar range is observed southwards, in the region comprised between Lake Kyoga, Lake Victoria, Lake Albert and the eastern borders with Kenya. South-westwards, infection probability increases following a positive gradient from γ≈0.30 to γ≈0.70 in the most southern districts (Figure 2B).

**Table 4:** Infection risk model results.

**Coefficient**	**Estimate**	**SE**	***p-value***	**OR^a^**	**OR_low_^b^**	**OR_up_^c^**
β${}_{0}^{\text{d}}$	−1.128	0.115	1.21E−22^***^	0.324	0.258	0.406
log_10_(*ψ_*R*_*)	−0.219	0.105	3.72E−02^*^	0.803	0.654	0.987
BIO_5_	−0.432	0.104	3.18E−05^***^	0.649	0.529	0.796
log_10_(Cd)	0.015	0.105	8.86E−01	1.015	0.826	1.247
log_10_(*ψ_*S*_*)	0.246	0.111	2.67E−02^*^	1.279	1.029	1.590

Point estimates (Estimate) of the standardized regression coefficients (Coefficient) are reported on the logit scale together with their standard errors (SE), p-values and the associated odds ratios (OR). Significant regression coefficients are highlighted with

*** when their p-values (p) are ≤ 0.001; ^**^when 0.001 < p ≤ 0.01;

* *when 0.01 < p ≤ 0.05*.

a *Odds ratios associated with regression coefficients express the expected change in the ratio γ/(1–γ), for a one standard deviation increase of the concerned predictor, holding all the other predictors fixed at a constant value*.

b *Odds ratio 95% confidence interval (CI), lower bound*.

c *Odds ratio 95% CI, upper bound.^d^Population intercept*.

### Landscape genomics

#### Population structure analysis

After pruning for MAF, LD, genotype and individual call rates, PSD counted 12,925 SNPs and 1,355 individuals, among which 743 from Uganda, 131 European taurine, 158 African taurine, 195 sanga from outside Uganda, and 128 zebu cattle.

Sanga and zebuine ancestries were the most represented in Uganda. Particularly, on average the sanga component constituted 76% (±13%) of the individual ancestries, whereas the zebuine counted 18% (±13%), with more than half of the individuals showing a zebuine proportion >20%. Further, ~3% of African and European taurine genomic ancestry components was also observed (Supplementary Figure 4). Genomic components showed spatial structure, the zebuine gene pool being more present in the North-East of the country, and the sanga in central and south-western Uganda (Supplementary Figure 10) (Stucki et al., 2017). The African taurine ancestry component was detectable as background signal especially in the North-West and South-West, whereas European introgression was mostly observed in the South-West.

The first three principal components (PC_1_, PC_2_ and PC_3_, respectively) explained almost the totality of the variance within admixture Q-scores for *K*=4; PC_1_ split the dataset between sanga and zebu gene pools, and PC_2_ and PC_3_ identified the European and African taurine components, respectively. Thus, these three PCs were used as population structure predictors to account for population structure within LGD in the landscape genomics models.

#### Genotype-environment associations

After QC, LGD counted 40,886 markers and 743 animals (the same in PSD) from 199 farms (4 ± 1 samples/farm), over 51 cells (15 ± 5 samples/cell).

**Figure 3:**
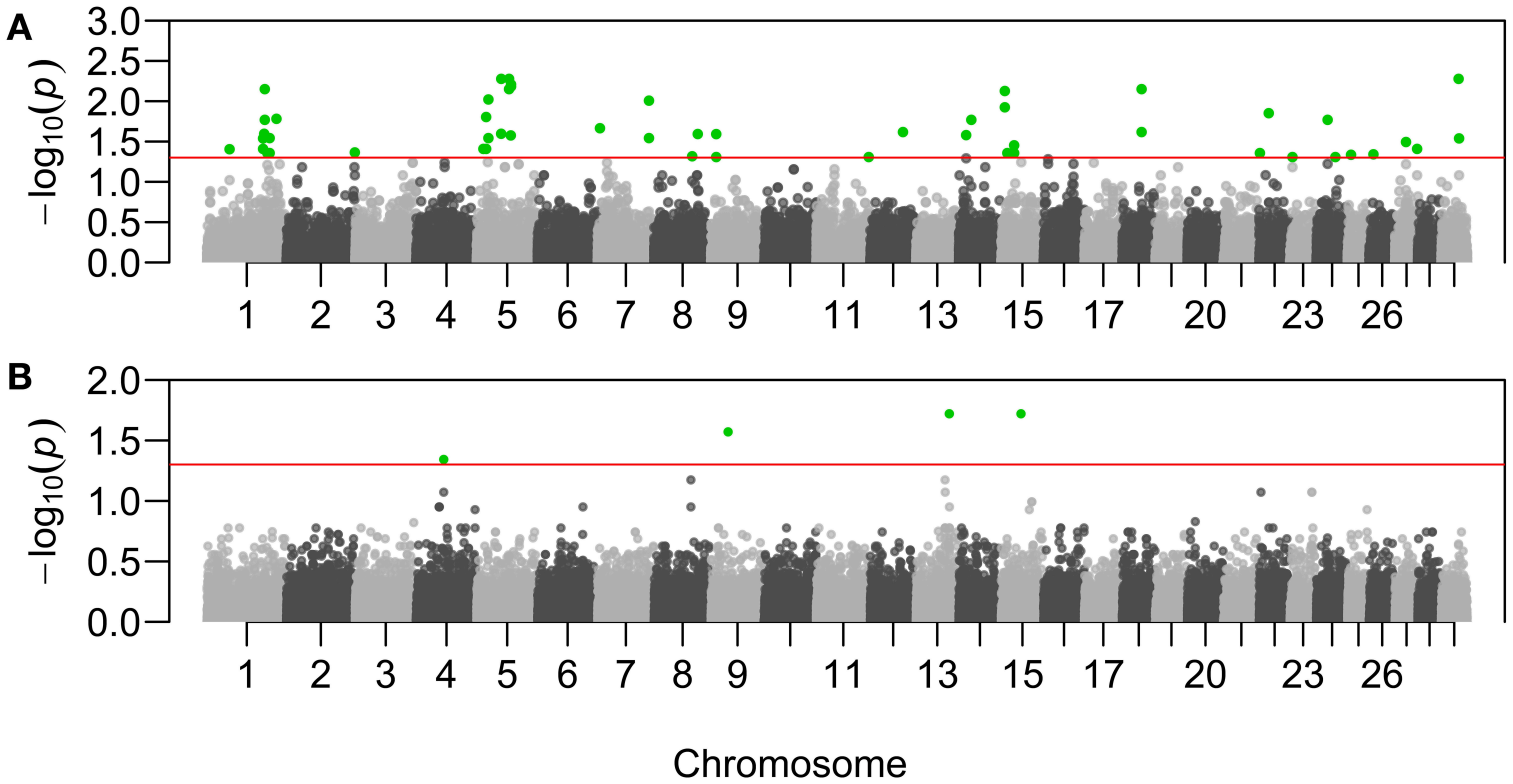
Manhattan plots of the genotype-environment associations. *X*-axis reports chromosomal position of the tested SNPs on *B. taurus* chromosomes. *Y*-axis reports the test statistic *p-values* (*p*) for the associations with *Rhipicephalus appendiculatus* occurrence probability **(A)**, and with *Theileria parva parva* infection risk **(B)**. *P-values* are displayed for each genotype after the Benjamini-Hochberg (BH) correction, and on the –log_10_ scale. Nominal significance threshold (α_BH_=0.05) is displayed as a red line, and significant *p-values* are highlighted in green.

Sixty-three genotypes across 41 putative adaptive loci resulted significantly associated with ψ_*R*_ (Figure 3A, Supplementary Table 2, and Supplementary Figures 11–12). Eight genotypes across seven loci resulted significantly associated with γ (Figure 3B, Supplementary Table 3, and Supplementary Figures 11–12).

#### Gene annotation

Among the 41 loci significantly associated with ψ_*R*_, 18 presented at least one annotated gene in the Ensembl database in close proximity (Table 5A and Supplementary Figure 12). Locus BTA-113604-no-rs (hereafter BTA-113604) is located ~12.5 kbp apart from the Protein kinase, cGMP-dependent, type I (*PRKG1*) gene on chromosome 26. *PRKG1* was already proposed as a candidate gene for tick resistance in South African Nguni cattle (Mapholi et al., 2016).

**Table 5:** Gene annotation for the loci significantly associated with ψ_*R*_ (A) and γ (B).

**SNP ID**	**Genotype(s)**	**Chr**.	**Position**	**Annotated gene**	**Biological function**
**A**					
ARS-BFGL-NGS-110339	AA,AC	1	111,495,891	Uncharacterized (111,445,583–111,512,320)	-
Hapmap34409-BES7_Contig244_858	AA	1	120,149,924	Glycogenin-1 (*GYG1*; 120,090,467–120,127,892)	Energy metabolism and angiogenesis (Lancaster et al., 2014)
Hapmap34056-BES2_Contig421_810	AG,GG	1	138,178,130	DnaJ heat shock protein family (Hsp40) member C13 (*DNAJC13*; 138,139,496–138,305,752)	Heat shock proteins (Kodiha et al., 2012)
ARS-BFGL-NGS-32909	CC,AC	5	67,846,632	5′-nucleotidase domain containing 3 (*NT5DC3*; 67,791,379–67,850,986)	UP-regulated genes for iron content in Nelore cattle (Wellison Jarles da Silva, 2015)
				Uncharacterized (67,852,917–67,930,472)	–
ARS-BFGL-NGS-37845	AG,AA	5	48,633,731	Methionine sulfoxide reductase B3 (*MSRB3*; 48,563,806–48,743,354)	Affect ear floppiness and morphology in dogs (Boyko et al., 2010)
BTA-46975-no-rs	CG,GG	5	68,220,538	Thioredoxin reductase 1. cytoplasmic (*TXNRD1*; 68,239,611–68,302,678)	Milk production and oocyte developmental competence in cattle (Gilbert et al., 2012; Ghorbani et al., 2015)
Hapmap51626-BTA-73514	AA,AG	5	48,834,486	Inner nuclear membrane protein Man1 (*LEMD3*; 48,773,272–48,844,474)	Height in pigs and cattle (Frantz et al., 2015)
UA-IFASA-6140	AG,AA	7	102,472,846	ST8 alpha-N-acetyl-neuraminide alpha-2.8-sialyltransferase 4 (*ST8SIA4*; 102,456,175–102,555,855)	Metabolism of milk glycoconjugates in mammals (Song et al., 2016)
BTB-00292673	AA	7	4,953,801	Phosphodiesterase 4C (*PDE4C*; 4,927,816–4,939,026)	Fertility (Glick et al., 2011)
				Member RAS oncogene family (*RAB3A*; 4,944,325–4,950,010)	Calcium exocytosis in neurons (Brondyk et al., 1995)
				MPV17 mitochondrial inner membrane protein like 2 (*MPV17L2*; 4,950,069–4,953,210)	Immune system (Brütting et al., 2016)
Hapmap31116-BTA-143121	AA	8	75,973,285	Epoxide hydrolase 2 (*EPHX2*; 75,908,165–75,977,482)	*In vitro* maturation. fertilization and culture on bovine embryos (Smith et al., 2009)
				L-gulonolactone oxidase (*GULO*; 75,984,696–76,010,699)	Involved into vitamin C production in pigs (Hasan et al., 2004)
ARS-BFGL-NGS-104610	AG	11	104,293,559	Surfeit 6 (*SURF6*; 104,296,135–104,302,894)	Housekeeping gene (Magoulas et al., 1998)
				Mediator complex subunit 22 (*MED22*; 104,305,076–104,311,650)	Gestation length in Nelore cattle (Matos et al., 2013)
				Ribosomal protein L7a (*RPL7A*; 104,311,808–104,315,125)	Oocyte developmental competence in cattle (Gilbert et al., 2012)
				Uncharacterized (104,315,458–104,334,584)	-
				Small nucleolar RNA (*SNORD24*; 104,312,993–104,313,063)	May act as methylation guide for RNA targets (Kiss-László et al., 1996)
				Small nucleolar RNA (*SNORD36*; 104,314,558–104,314,622)	2'-O-ribose methylation guide (Galardi et al., 2002)
				Small nucleolar RNA (*snR47*; 104,313,768–104,313,828)	2'-O-methylation of large and small subunit rRNA (Samarsky and Fournier, 1999)
				Small nucleolar RNA (*SNORD24*; 104,312,260–104,312,334)	As above
				Small nucleolar RNA (*SNORD36*; 104,314,159–104,314,231)	As above
BTB-00839408	AG. AA	22	18,978,658	Metabotropic glutamate receptor 7 precursor (*GRM7*; 18,740,484–19,647,747)	Might be related to parasite resistance (Xu et al., 2016)
ARS-BFGL-NGS-39898	GG	22	1,319,636	Novel gene (1,310,943–1,311,505)	–
ARS-BFGL-BAC-31319	AA	23	4,847,028	3-hydroxymethyl-3-methylglutaryl-CoA lyase like 1 (*HMGCLL1*; 4,7 09,297–4,906,605)	Involved into ketogenesis (Tetens et al., 2015)
Hapmap51155-BTA-11643	AA	24	38,086,180	DLG associated protein 1 (*DLGAP1*; 37,994,546–38,293,883)	Role in neurological development and behavioral disorders (Sorbolini et al., 2015)
Hapmap57868-rs29020458	AA	24	22,746,291	Dystrobrevin alpha (*DTNA*; 22,445,691–22,767,026)	Formation and stability of synapses (Sjö et al., 2005)
				U6 spliceosomal RNA (*U6*; 22,759,777–22,759,879)	Participate into spliceosome formation (Marz et al., 2008)
BTA-113604-no-rs	AA	26	8,356,096	Protein kinase. cGMP-dependent. type I (*PRKG1*; 6,906,081–8,343,629)	Tick resistance in South African Nguni cattle (Mapholi et al., 2016)
ARS-BFGL-NGS-18933	GG	29	34,650,967	Opioid binding protein/cell adhesion molecule like (*OPCML*; 34,554,780–35,085,038)	Role in opioid receptor function in humans (Smith et al., 1993)
**B**					
BTB-01298953	AA	4	54,930,726	Protein phosphatase 1 regulatory subunit 3A (*PPP1R3A*; 54,866,421–54,906,096)	Glycogen synthesis in humans and mice (Savage et al., 2008)
BTA-33234-no-rs	GG	13	66,291,997	DLG associated protein 4 (*DLGAP4*; 66,204,671–66,292,988)	Neuronal membrane protein (Takeuchi et al., 1997)
				Myosin light chain 9 (*MYL9*; 66,306,260–66,314,230)	May participate in regulation of muscle contraction (Kumar et al., 1989)
ARS-BFGL-NGS-112656	AA	13	66,336,246	Myosin light chain 9 (*MYL9*; 66,306,260–66,314,230)	As above
				TGFB induced factor homeobox 2 (*TGIF2*; 66,334,680–66,351,481)	Transcriptional repressor (Imoto et al., 2000)
ARS-BFGL-NGS-110102	GG	13	66,370,867	TGFB induced factor homeobox 2 (*TGIF2*; 66,334,680–66,351,481)	As above
				TGIF2-C20orf24 readthrough (*C13H20orf24* alias *RIP5*; 66,362,562–66,369,978)	May promote apoptosis in humans (Zha et al., 2004)
				Src-like-adaptor 2 (*SLA*2; 66,368,694–66,395,549)	Downregulation of T and B cell-mediated responses (Holland et al., 2001)
ARS-BFGL-NGS-24867	AA	13	66,395,465	Src-like-adaptor 2 (*SLA*2; 66,368,694–66,395,549)	As above
				NDRG family member 3 (*NDRG3*; 66,398,147–66,594,149)	Linked to prostate cancer cells growth (Lee et al., 2016)
Hapmap39482-BTA-36746	CC,AC	15	40,279,014	TEA domain transcription factor 1 (*TEAD1*; 40,303,805–40,482,346)	Transcription, factor promoting apoptosis in mammals (Landin Malt et al., 2012)

*Single-nucleotide polymorphisms in linkage disequilibrium with genes annotated in the Ensembl database are reported with the genotype(s) originally highlighted by SAMHβADA, the membership chromosome (Chr) and physical position in base pairs on the chromosome, as well as the names and biological function of the annotated genes, as found for a reference species. Physical position of the annotated genes is reported in brackets after the gene names (fifth column)*.

Six out of the seven loci significantly associated with γ presented at least one annotated gene within the selected window size (Table 5B and Supplementary Figure 12). Two SNPs (ARS-BFGL-NGS-110102 and ARS-BFGL-NGS-24867, hereafter ARS-110102 and ARS-24867, respectively) were proximal to the Src-like-adaptor 2 (*SLA*2) gene on chromosome 13. *SLA*2 human orthologue encodes the Src-like-adaptor 2, a member of the SLAP protein family which regulates the T and B cell-mediated immune response (Holland et al., 2001). Given *T. p. parva* known ability to promote the proliferation of T and B cells (Baldwin et al., 1988; Dobbelaere and Küenzi, 2004), we considered *SLA*2 as a second candidate gene for ECF local adaptation.

### Local ancestry

#### PCAdmix analysis

Based on the gene pools revealed by Admixture analysis in Ugandan indigenous cattle, we performed PCAdmix analysis using one zebuine (Tharparkar; THA) and one African taurine (Muturu; MUT) reference (Supplementary Text 4). After QC, LAD counted 689,339 markers and 128 individuals (102 Ugandan cattle individuals, 13 THA, and 13 MUT).

For the genomic window hosting BTA-113604 (i.e., window 13 on chromosome 26; Supplementary Figure 13), 79 out of the 204 haploid individuals targeted showed MUT ancestry, while 125 THA ancestry (Supplementary Figure 14). For the genomic window hosting ARS-110102 and ARS-24867 (i.e., window 145 on chromosome 13; Supplementary Figure 13), 63 haploid individuals were assigned to MUT, while 141 to THA (Supplementary Figure 14).

#### Beta regression analysis

**Figure 4:**
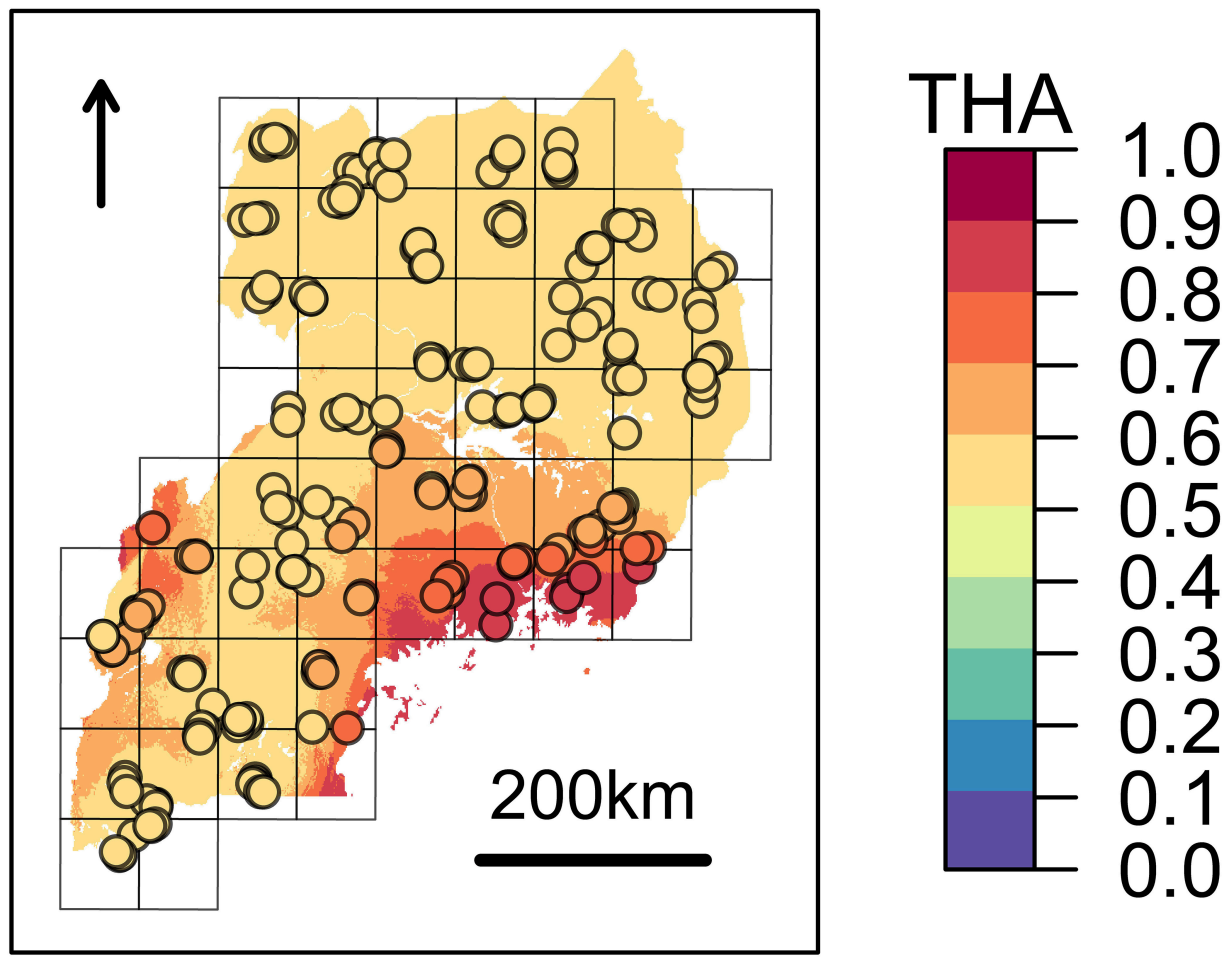
Expected zebuine proportion of the genomic region candidate for tick resistance. The association inferred through beta regression between Tharparkar ancestry (THA) and average *Rhipicephalus appendiculatus* occurrence probability per cell (Table 6) was used to generalize expected zebuine ancestry over Uganda. Colour key corresponds to predicted THA proportion, with increasing values from the blue to the red tones. Sampled farms are represented with circles, and coloured according to the predicted THA proportion at their geographical location.

Tharparkar ancestry at window 13 of chromosome 26 showed a positive and significant association with ψ_*Rc*_ (*H*_0_: β_*i*_=0, α=0.05) (Table 6 and Figure 4), while no significant association was found between the Muturu/Tharparkar ancestries at window 145 of chromosome 13 and γ_*c*_ (Supplementary Text 5).

**Table 6:** Beta regression results.

**Coefficient**	**Estimate**	**SE**	***p-value***	**OR^a^**	**OR_low_^b^**	**OR_up_^c^**
β_0_	0.144	0.194	4.56E−01	1.155	0.790	1.689
*ψ_*Rc*_*	1.663	0.768	3.04E−02^*^	5.275	1.171	23.767
ϕ	2.029	0.346				

Association between the inferred proportion of THA ancestry at window 13 (chromosome 26) with average Rhipicephalus appendiculatus occurrence probability per sampling cell (ψ_Rc_). Point estimates (Estimate) of the intercept (β_0_), the regression coefficient associated to ψ_Rc_ and the precision parameter ψ are reported on the logit scale together with their standard errors (SE). P-values and odds ratios (OR) are shown for β_0_ and ψ_Rc_. Significant regression coefficients are highlighted with ***when their p-values (p) are ≤ 0.001; **when 0.001 < p ≤ 0.01;

* *when 0.01 < p ≤ 0.05*.

a *Odds ratio 95% confidence interval (CI), lower bound*.

b *Odds ratio 95% CI, upper bound*.

## Discussion

East Coast fever represents a major issue for livestock health in sub-Saharan countries (Nene et al., 2016), with over one million cattle deceased every year, and an annual economic damage of 168–300 million USD (Norval et al., 1992; McLeod and Kristjanson, 1999).

ECF incidence is highly correlated with the geographical distribution of the tick vector *R. appendiculatus*, whose occurrence is an essential precondition for *T. p. parva* infection in cattle (Olwoch et al., 2008). However, with the present study we show that areas with predicted poor habitat suitability for the tick can present higher infection rates when compared with regions highly suitable for the tick (Figure 2 and Table 4). Such observation suggests that additional factors may contribute in explaining the observed *T. p. parva* infection patterns; here, we suggest three possible hypotheses.

First, environmental temperature may play a pivotal role in defining *T. p. parva* infection risk. Piroplasm development within the tick vector appears to be hindered by temperatures >28°C persisting even for short time periods (as less as 15 days) (Young and Leitch, 1981). Therefore, areas exceeding this temperature threshold might present a reduced infection risk due to the low success in parasite development and transmission. The presence of such a temperature constraint might concur in explaining the low infection risk predicted in the regions such as North-East of Lake Victoria, where a highly suitable habitat is predicted for *R. appendiculatus*, but where temperature can reach 30°C in the warmest month of the year (January) (Hijmans et al., 2005). Coherently, in the south-western area, environmental temperature ranges between ~8 and 28°C during the whole year (Hijmans et al., 2005), and the predicted risk of infection increases possibly reflecting a higher efficiency in the parasite transmission despite the predicted decrease in tick occurrence probability.

Second, the most suitable areas for the vector (Figure 2A) overlap those regions where the highest levels of zebuine ancestry were recorded (Supplementary Figure 10). *B. t. indicus* is known to be more effective in counteracting tick infestation than *B. t. taurus* (Brizuela et al., 1996; Wambura et al., 1998; Mattioli et al., 2000; Jonsson et al., 2014), and is consequently less affected by tick-borne micro-organisms (Mattioli et al., 2000), including *T. p. parva*, whose effects are known to be dose-dependent (Brossard and Wikel, 1997; Nene et al., 2016). The core adaptive response to tick burden was identified as the inflammatory reaction triggered by the tick bite at the cutaneous level (Mattioli et al., 2000), which activates a strong white cells-mediated cutaneous reaction (Willadsen, 1980) affecting attachment, salivation, engorgement, and ultimately limiting the inoculation of tick-borne microorganisms (Wikel and Bergrnan, 1997). Therefore, the low infection risk observed in the most suitable areas for *R. appendiculatus* (e.g., north-eastern districts) might be explained by the coexistence of putative tick-resistant zebuine-like populations (Bahbahani et al., 2017), along with a sub-optimal environmental niche for the parasite. Further, we speculate that cattle populations living in regions suitable for *T. p. parva* development, but with reduced predicted tick burden (e.g., the southern districts; Figures 2A,B), could have not undergone a tick-specific adaptation, and therefore show higher infection rates.

Third, the *R. appendiculatus* distribution model does not explicitly consider anthropogenic factors like tick-control campaigns on a local and temporal basis. However, adequate tick-control campaigns are rarely undertaken in Uganda (Ugandan National Drug Authority), and evidence of *R. appendiculatus* developing drug resistance has been recorded (Vudriko et al., 2016).

Despite *T. p. parva* infection being observed in the northern farms of Uganda, a low tick occurrence probability is predicted for the same regions (ψ_*R*_<0.1; Figure 2A). A possible explanation is the lack of *R. appendiculatus* records from these areas, and the consequent bias in the tick distribution model (Cumming, 1999b; Olwoch et al., 2003). Moreover, predicted infection risk in the North (γ<0.3; Figure 2B) may be inflated by the inverse relationship between γ and ψ_*R*_ as estimated by the infection risk model (Table 4), and care is recommended regarding the infection risk predictions for these areas.

Local adaptation is prone to evolve in host-parasite systems, given the strong (and often reciprocal) selection imposed by one species to the other, the reduced role of phenotypic plasticity, and the small number of genes with strong effects usually involved (Kawecki and Ebert, 2004). In a spatial context, a gradient of selection intensity (i.e., spatially varying selection) is required over the landscape, with coexistence areas showing higher selective pressure being candidate for local adaptive responses to evolve. In the present case, host distribution encompasses regions having different selective pressure in terms of both tick and parasite burdens (Figures 2A,B). Regions with higher *T. p. parva* selective pressure (implying tick occurrence with effective transmission) are those where the host population is expected to be locally adapted to infection; whereas, regions with higher tick burden (but lower infection risk) are those where tick-resistant populations are expected to occur. Following this rational, we suggest that the putative adaptive component sustaining ECF-tolerance/resistance might be due to a synergic mechanism involving specific adaptations to *R. appendiculatus* and *T. p. parva*.

Specifically, adaptations to tick burden could be found along the Lake Victoria coasts, where a higher selective pressure linked to *R. appendiculatus* is predicted (Figure 2A). We identified 41 loci across 18 chromosomes significantly associated with ψ_*R*_ (Figure 3A), with the majority of putative loci under selection found on chromosomes 5 (nine loci), 1 (seven loci), and 15 (three loci). Interestingly, the large genomic region hosting the associated SNPs on chromosome 5 (Supplementary Table 2) overlaps a genomic region which has been previously associated with several traits in tropical cattle, including parasite resistance (Porto-Neto et al., 2014). Among the genes in LD with the associated markers, we found *PRKG1* on chromosome 26 (Table 5A and Supplementary Figure 13), a gene coding for an important mediator of vasodilation, and already reported as possibly involved in tick resistance in the South African Nguni breed (see Table 6 in Mapholi et al., 2016). Importantly, vasodilatation is a classical feature of the inflammatory response (Sherwood and Toliver-Kinsky, 2004; Surks, 2007), the core mechanisms underlying tick resistance, as discussed before. None of the remaining annotated genes was easily attributable to adaptation to tick burden (Table 5A).

A specific adaptive response toward *T. p. parva* infection may have evolved in south-western Uganda, possibly due to ecological conditions more suitable for parasite survival and successful transmission, and the presence of a more tick-susceptible cattle population (Supplementary Figure 10). *Theileria parva* pathogenicity is linked to its ability to invade host lymphocytes, and promoting their transformation and clonal expansion through the activation of several host-cell signalling pathways (McKeever and Morrison, 1990; Dobbelaere and Küenzi, 2004; Chaussepied et al., 2010). Here, we found seven markers significantly associated with γ, two of which (ARS-110102 and ARS-24867) included within *SLA*2 genic region on chromosome 13 (Supplementary Figure 13). *SLA*2 is known to be involved with signal transduction in B and T cells, playing a role into downregulation of humoral and cell-mediated immune responses, and thus contributing to a correct activation and proliferation of lymphocytes (Holland et al., 2001; Kazi et al., 2015; Marton et al., 2015). *SLA2* antagonistic effect on lymphocytes proliferation would suggest its putative involvement in opposing the diffusion of *T. p. parva* in the organism.

Replication studies performed in areas with analogous host characteristics and selective gradients, like Kenya or Tanzania (Giblin, 1990; Gachohi et al., 2012; Laisser et al., 2017), and following the same methodology applied here would allow to validate the generality of the adaptive patterns highlighted, as well as to further control for the confounding effects of population structure and unconsidered collinear environmental features (Rellstab et al., 2015). Further, experimental validation will be essential to finally verify the physiological effect of the identified genes, and thus considering targeted breeding schemes.

Despite the genetic proximity between Muturu and some tick resistant indigenous *B. t. taurus* breeds of western Africa (i.e., N'Dama) Mattioli et al., 2000; Ibeagha-Awemu et al., 2004, local ancestry of the genomic region candidate for tick resistance was predominantly assigned to Tharparkar (Figure 4, Table 6, and Supplementary Figure 14). This result is in agreement with the known resistance of zebuine cattle to ticks, and suggests the origin of tick resistance in eastern Africa either from imported Indian populations or within local zebuine-like populations after migration from India. Conversely, no easily-interpretable indication was obtained for the genomic region candidate for tolerance to *T. p. parva* infection. Indeed, neither Tharparkar nor Muturu ancestries displayed a significant association with infection risk, while an additional local ancestry analysis revealed a positive correlation with the European taurine Hereford ancestry when tested versus Tharparkar (Supplementary Text 5). Although surprising, this result would rather point toward a taurine origin of infection tolerance/resistance. However, local ancestry results are inherently reference-dependent (Barbato et al., 2017), and further analyses with different African taurine and zebuine references will be required to disentangle the evolutionary origin of the genomic regions under scrutiny.

Besides the identification of candidate regions for local adaptation, our results revealed allochthonous introgression from Europe within the local gene pools of indigenous Ugandan *B. taurus* populations (Supplementary Text 4 and Supplementary Figure 10). This finding is consistent with the generalized loss of agro-biodiversity reported worldwide (FAO, 2015; Mwai et al., 2015), and stresses the importance of monitoring local genetic resources to conserve unique adaptations, including tolerance and/or resistance to tropical endemic diseases.

Despite limitations in both epidemiological and species occurrence data, the proposed methodological framework allowed the identification of two candidate genes putatively associated with local adaptation to East Coast fever. Overall, the combination of ecological modelling (i.e., species distribution and infection risk models) and landscape genomics showed the potential of detecting candidate genomic regions showing adaptive significance, and can assist in unravelling the adaptive patterns underlying any kind of symbiotic relationship like host-parasite interactions, mutualism, and commensalism, as well as competition among species.

## Data accessibility

The datasets generated and analyzed for this study, as well as the source code developed for data analysis, can be found in the Dryad Digital Repository (doi: 10.5061/dryad.sf5j2bf). Raster data are available from the public sources mentioned in the references and in Supplementary Text 1.

## Author contributions

EV, MB, LC, MM, ER, EF, MD, RN, SJ, and PA-M: Conceptualization; EV, MB, LC, and MM: Data curation; EV and MB: Formal analysis; EV, MB, ER, EF, SJ, and PA-M: Methodology; EV, MB, MM, and MD: Software; EV: Visualization, original draft preparation; MB, LC, MM, ER, SJ, TSS, and PA-M: Review and editing; LC, MM, FK, and RN: Investigation; LC, RN, SJ, and PA-M: Funding acquisition; LC, SJ, and PA-M: Supervision; SJ and PA-M: Project administration; CMu, CMa, VBM, FK, TSS, and HJH: Resources.

### Conflict of interest statement

TSS was employed by company Recombinetics, Inc. The remaining authors declare that the research was conducted in the absence of any commercial or financial relationships that could be construed as a potential conflict of interest.

## Footnotes

^1^US Geological Survey (USGS). Earth Resources Observation and Science (EROS) Center - Famine Early Warning Systems Network (FEWS NET). Available at: https://earlywarning.usgs.gov/fews.

^2^http://nextgen.epfl.ch

## Abbreviations

Cd: Cattle density
*D*: Likelihood-ratio test statistic
|r|: Absolute Pearson's product-moment correlation coefficient
r^2^: Global linkage disequilibrium decay
γ: *Theileria parva parva* infection risk in cattle
γ_*c*_: Average γ in the *c* sampling cell
π_*i*_: Genotype occurrence probability in the *i-th* individual
ψ: Species occurrence probability
ψ_*x*_: Species occurrence probability in the *x* pixel of the landscape
ψ_*R*_: *Rhipicephalus appendiculatus* occurrence probability
ψ_*Rx*_: *Rhipicephalus appendiculatus* occurrence probability in the *x* pixel of the landscape
ψ_*Rc*_: Average ψ_*R*_ in the *c* sampling cell
ψ_*S*_: *Syncerus caffer* occurrence probability
ψ_*Sx*_: *Syncerus caffer* occurrence probability in the *x* pixel of the landscape.
