# Supplementary material for: Combining Landscape Genomics and Ecological Modelling to Investigate Local Adaptation of Indigenous Ugandan Cattle to East Coast Fever
Source: Front Genet. 2018 Oct 3;9:385. doi: 10.3389/fgene.2018.00385 (PMC6177531; doi:10.3389/fgene.2018.00385)
Supplement: Supplementary file 1 [file Presentation_1.pdf]

Supplementary Material

Combining landscape genomics and ecological modelling to investigate local adaptation of indigenous Ugandan cattle to East Coast fever

Elia Vajana[*], Mario Barbato, Licia Colli, Marco Milanesi, Estelle Rochat, Enrico Fabrizi, Christopher Mukasa, Marcello Del Corvo, Charles Masembe, Vincent Muwanika, Fredrick Kabi, Tad Stewart Sonstegard, Heather Jay Huson, Riccardo Negrini, Stéphane Joost, Paolo Ajmone-Marsan, on behalf of The NextGen Consortium

*** Correspondence:** Elia Vajana: elia.vajana@epfl.ch

1 Supplementary Text

1.1 Supplementary Text 1. MODIS collection 5 data.

The 72 ten-days annual NDVI periods used in the present study are based on the MODIS collection 5 (C5) data, whose processing ended in March 2017 (Budde, 2018; *personal communication*). C5 data are no longer distributed, so that the link http://earlywarning.usgs.gov/fews/product/116 originally used for the download has expired. Upon permission from the US Geological Survey (USGS), we make available the C5 NDVI data from the Dryad Digital Repository (Heneghan et al., 2011): doi:10.5061/dryad.sf5j2bf.

1.2 Supplementary Text 2. Statistical significance of genotype-environment associations.

Statistical significance of genotype-environment associations was evaluated by means of a likelihood-ratio (LR) test. Null and alternative models (NM and AM, respectively) were compared for each genotype.

Given a specific genotype, NM was specified as:

$$ln\left(\frac{\pi_i}{1-\pi_i}\right) = \beta_0 + \sum_{s=1}^{S} \beta_s x_{si}$$

where β_0 represents the model intercept, β_s the regression coefficient related to the *s-th* population structure predictor, and x_{si} the *i-th* observation of the *s-th* population structure predictor.

AM was specified as:

$$ln\left(\frac{\pi_i}{1-\pi_i}\right) = \beta_0 + \beta_Z z_i + \sum_{s=1}^{S} \beta_s x_{si}$$

where β_Z is the regression coefficient for the environmental variable Z, and z_i is the *i-th* observation of Z.

This way, NM is nested within AM (i.e. NM=AM when β_Z=0). Then, a LR test was performed for each genotype to test if the inclusion of Z (i.e. in turn ψ_R or γ) led to a significantly improved explanation of the genotype spatial distribution. Particularly, as SAMβADA returns log-likelihood (LogL) values, the LR test was specified as:

$$D = -2(\text{LogL NM} - \text{LogL AM})$$

Under the null hypothesis that NM and AM have the same log-likelihoods (i.e. D=0), D follows a χ^2 distribution with degrees of freedom equal to the difference in the number of parameters between AM and NM. Here, *p-values* were derived from a χ^2 distribution with one degree of freedom (AM having one parameter more than NM; see figure hereafter). *P-values* estimate was performed with the R function `pchisq`, by setting the appropriate value for degrees of freedom. The option `lower=FALSE` was used to compute the probability of obtaining the observed (or more extreme) D values under the null hypothesis.

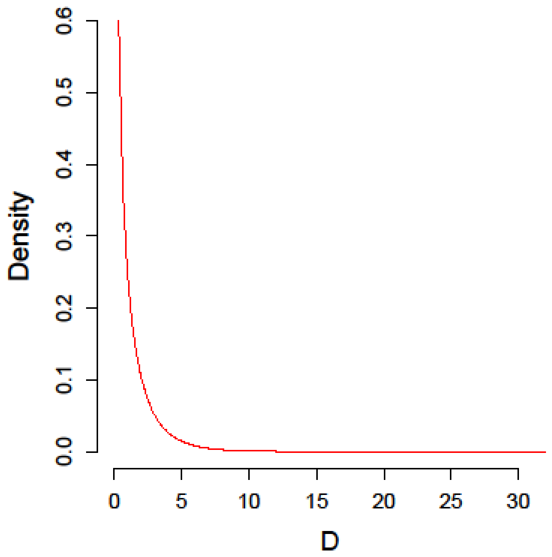

Expected distribution of the likelihood-ratio test statistic (*D*) under the null hypothesis that NM and AM have the same log-likelihoods (i.e. *D*=0). Here, *D* values are distributed following a χ^2 distribution with one degree of freedom.

1.3 Supplementary Text 3. Additional SAMβADA analysis (*K*=3 and *K*=16 corrections).

In order to test SAMβADA results obtained with the *K*=4 correction (see main text), we also performed genotype-environment associations tests with alternative population structure predictors. Particularly, an additional ADMIXTURE analysis was performed on PSD for *K* values from two to 30 (Supplementary Figure 4) (Kopelman et al., 2015), and the membership coefficients from the *K*=3/*K*=16 cluster solutions were independently used as population structure predictors: *K*=3 was chosen to account for the fundamental gene pools present in cattle (i.e. African *Bos taurus taurus*, European *B. t. taurus* and *B. t. indicus*), and *K*=16 as the cluster solution showing the lowest CV error (see figure hereafter). In both the cases, a PCA was performed on the membership coefficients to obtain synthetic and orthogonal population structure predictors following the same procedure described in main text. When correcting for *K*=3, the first and second principal components (accounting for 63% and 37% of the variance within membership coefficients, respectively) were retained. The principal components from one to seven (accounting for 70% of the original variance within membership coefficients) were used when correcting for *K*=16 (Jolliffe, 2002). Results of additional SAMβADA analysis for the *K*=3/*K*=16 corrections are reported in Supplementary Figure 12.

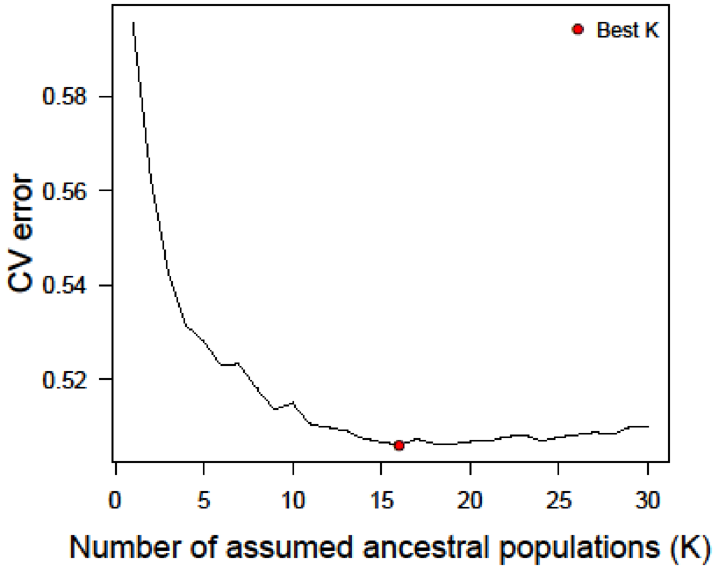

Cross-validation (CV) errors associated with the cluster solutions (*K*) tested for partitioning PSD.

1.4 Supplementary Text 4. Selection of reference populations for local ancestry analyses.

Global ancestry analysis evidenced the presence of all the fundamental cattle gene pools in the individuals composing LGD, i.e. *B. t. indicus*, African *B. t. taurus* and European *B. t. taurus*, other than a major admixed sanga component (see main text, and Supplementary Figure 4 for $K \geq 3$). Non-admixed reference populations were used for local admixture analysis, in order to potentiate PCADMIX discriminant power (Barbato et al., 2017). Therefore, we selected two zebuine references (Tharparkar and Lohani), one African *B. t. taurus* reference (Muturu), and one European *B. t. taurus* reference (Hereford; see table hereafter), all expected with null no or negligible signals of introgression according to global ancestry results (Supplementary Figure 4) and literature findings (Decker et al., 2014).

Four taurine/zebuine references combinations were tested: (i) Muturu-Tharparkar; (ii) Muturu-Lohani; (iii) Hereford-Tharparkar; (iv) Hereford-Lohani.

Reference populations used in PCADMIX analysis.

Reference population	Code	Type	N	Origin	Source
Tharparkar	THA	*B. t. indicus*	13	Asia (Pakistan)	S[a]
Lohani	LOH	*B. t.indicus*	13	Asia (Pakistan)	S
Muturu	MUT	African *B. t. taurus*	13	West Africa	S
Hereford	HFD	European *B. t. taurus*	27	Europe	S

The table summarizes breed names (Reference population), acronyms (Code), cattle type (Type), sample sizes (N), geographical origin (Origin), and data source (Source). [a]Genotypes provided by T. S. Sonstegard.

1.5 Supplementary Text 5. Beta regression results on additional local ancestry analyses.

Beta regression analysis was performed after all PCADMIX comparisons (Supplementary Text 4), by following the procedure described in the main text (see section Materials and Methods). Hereafter, results are shown for (i) the Muturu-Tharparkar comparison (Table 1), (ii) the Muturu-Lohani comparison (Table 2), (iii) the Hereford-Tharparkar comparison (Table 3), and (iv) the Hereford-Lohani comparison (Table 4).

Table 1. Muturu-Tharparkar comparison (result shown in the main text).

**Coefficient**	**Estimate**	**SE**	***p-value***	**OR**	**OR_{low}** [a]	**OR_{up}** [b]
β_0	0.144	0.194	4.56E−01	1.155	0.790	1.689
ψ_{Rc}	**1.663**	0.768	**3.04E−02***	5.275	1.171	23.767
ϕ	2.029	0.346				
**Coefficient**	**Estimate**	**SE**	***p-value***	**OR**	**OR_{low}**	**OR_{up}**
β_0	1.362	0.479	4.44E−03*	3.904	1.527	9.983
γ_c	−2.067	1.695	2.23E−01	0.127	0.005	3.509
ϕ	2.103	0.375				

Upper table: beta regression results between Tharparkar ancestry at window 13 (chromosome 26) and average *Rhipicephalus appendiculatus* occurrence probability per cell (ψ_{Rc}). *Lower table*: association between Tharparkar ancestry at window 145 (chromosome 13) and average infection risk per cell (γ_c), as derived from beta regression analysis. Point estimates of the intercept (β_0), the regression coefficients associated to ψ_{Rc} and γ_c, and the precision parameter ϕ are reported on the logit scale together with their standard errors (SE). *P-values* and odds ratios (OR) are shown for β_0, ψ_{Rc}, and γ_c. [a]Odds ratio 95% confidence interval (CI), lower bound. [b]Odds ratio 95% CI, upper bound. Significant regression coefficients are highlighted with *** when their *p-values* (p) are $\leq$0.001; ** when 0.001<$p\leq$0.01; * when 0.01<$p\leq$0.05; · when 0.05<$p\leq$0.1. Significant coefficients are represented in bold.

Table 2. Muturu-Lohani comparison.

**Coefficient**	**Estimate**	**SE**	***p-value***	**OR**	**OR_{low}**	**OR_{up}**
β_0	0.935	0.196	1.73E−06***	2.547	1.735	3.740
ψ_{Rc}	0.999	0.732	1.72E−01	2.716	0.647	11.401
ϕ	2.566	0.480				
**Coefficient**	**Estimate**	**SE**	***p-value***	**OR**	**OR_{low}**	**OR_{up}**
β_0	1.012	0.471	3.16E−02*	2.751	1.093	6.925
γ_c	−0.481	1.680	7.75E−01	0.618	0.023	16.640
ϕ	2.164	0.389				

Upper table: association between Lohani ancestry at window 13 (chromosome 26) and ψ_{Rc}, as derived from beta regression analysis. *Lower table*: association between Lohani ancestry at window 145 (chromosome 13) and γ_c, as derived from beta regression analysis. Column headings, acronyms and codes for statistical significance are the same described in Table 1.

Table 3. Hereford-Tharparkar comparison.

**Coefficient**	**Estimate**	**SE**	***p-value***	**OR**	**OR_{low}**	**OR_{up}**
β_0	−0.429	0.208	3.93E−02*	0.651	0.433	0.979
ψ_{Rc}	1.360	0.814	9.46E−02·	3.896	0.790	19.210
ϕ	1.585	0.261				
**Coefficient**	**Estimate**	**SE**	***p-value***	**OR**	**OR_{low}**	**OR_{up}**
β_0	1.879	0.483	1.00E−04***	6.547	2.540	16.872
γ_c	**−3.851**	1.693	**2.29E−02***	0.021	0.001	0.587
ϕ	2.205	0.399				

Upper table: association between Tharparkar ancestry at window 13 (chromosome 26) and ψ_{Rc}, as derived from beta regression analysis. *Lower table*: association between Tharparkar ancestry at window 145 (chromosome 13) and γ_c, as derived from beta regression analysis. Column headings, acronyms and codes for statistical significance are the same described in Table 1.

Table 4. Hereford-Lohani comparison.

**Coefficient**	**Estimate**	**SE**	***p-value***	**OR**	**OR_{low}**	**OR_{up}**
β_0	1.025	0.206	6.34E−07***	2.787	1.861	4.174
ψ_{Rc}	0.778	0.747	2.98E−01	2.177	0.504	9.413
ϕ	2.240	0.422				
**Coefficient**	**Estimate**	**SE**	***p-value***	**OR**	**OR_{low}**	**OR_{up}**
β_0	1.232	0.477	9.81E−03**	3.428	1.346	8.731
γ_c	−0.498	1.689	7.68E−01	0.608	0.022	16.650
ϕ	2.114	0.395				

Upper table: association between Lohani ancestry at window 13 (chromosome 26) and ψ_{Rc}, as derived from beta regression analysis. *Lower table*: association between Lohani ancestry at window 145 (chromosome 13) and γ_c, as derived from beta regression analysis. Column headings, acronyms and codes for statistical significance are the same described in Table 1.

2 Supplementary Figures and Tables

2.1 Supplementary Figures

2.1.1 Supplementary Figure 1. Bioclimatic variables used in *Rhipicephalus appendiculatus* distribution model.

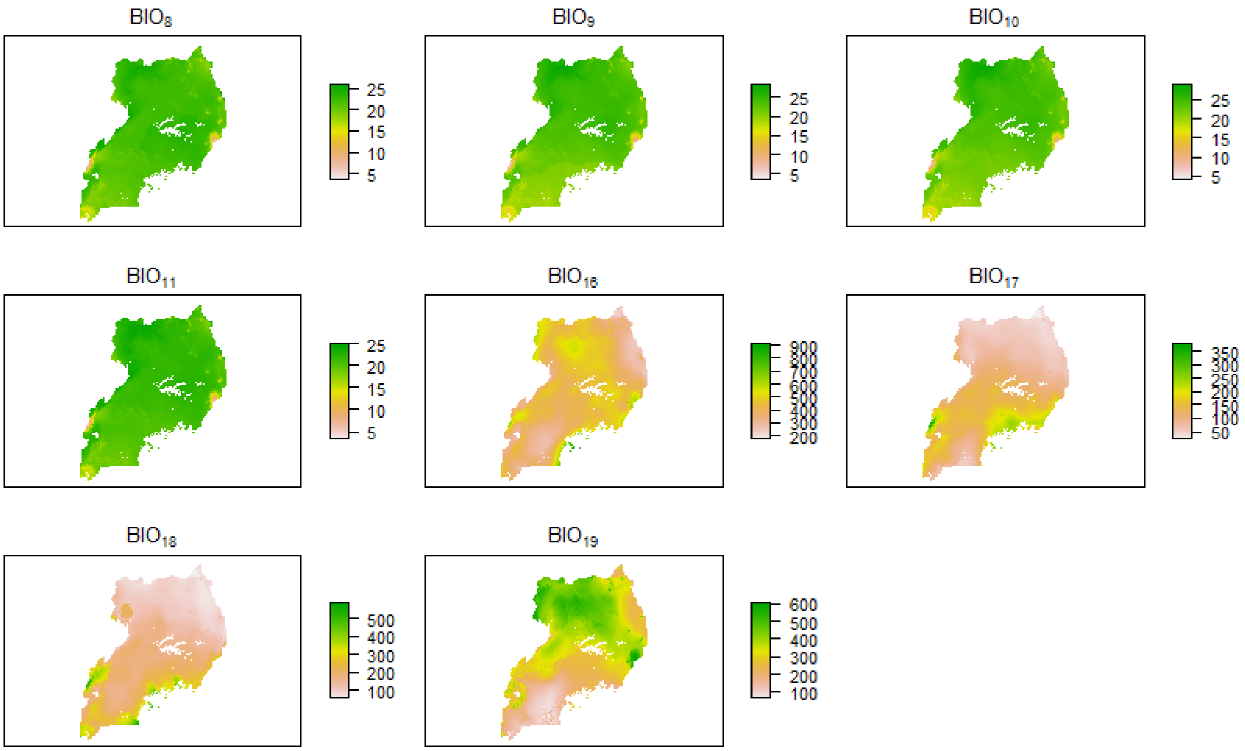

Bioclimatic variables used in *R. appendiculatus* distribution model. See Table 1 (main text) for details. BIO_8, BIO_9, BIO_{10}, BIO_{11} were transformed from decimal C° to C° prior analyses. BIO_{16}, BIO_{17}, BIO_{18}, BIO_{19} are expressed in millimetres.

2.1.2 Supplementary Figure 2. Selection of the annual period with NDVI values best explaining *Syncerus caffer* records.

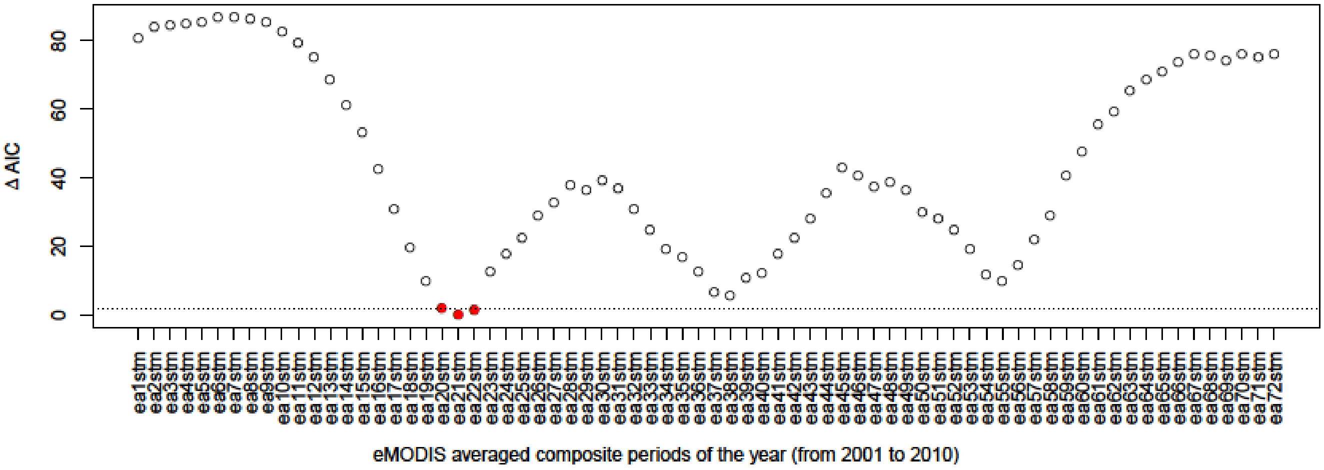

Seventy-two models were tested with Maxlike (Royle et al., 2012), one for each "eMODIS" annual period (or composite; see *X*-axis in the plot). Model performances were evaluated through the Akaike information criterion (AIC). Here, ΔAIC values (*Y*-axis) are plotted representing the differences between the AIC values of the tested models and the AIC value of the best model. The model testing composite 21 (ea21stm; April 6-15) resulted the best one displaying a ΔAIC=0. The horizontal dotted line corresponds to ΔAIC=2, which is a suggested threshold to discriminate models with a performance comparable to the best one (Muscarella et al., 2014). ΔAIC values of the best model and those <2 are highlighted in red.

2.1.3 Supplementary Figure 3. Outlier detection in the infection risk model predictors.

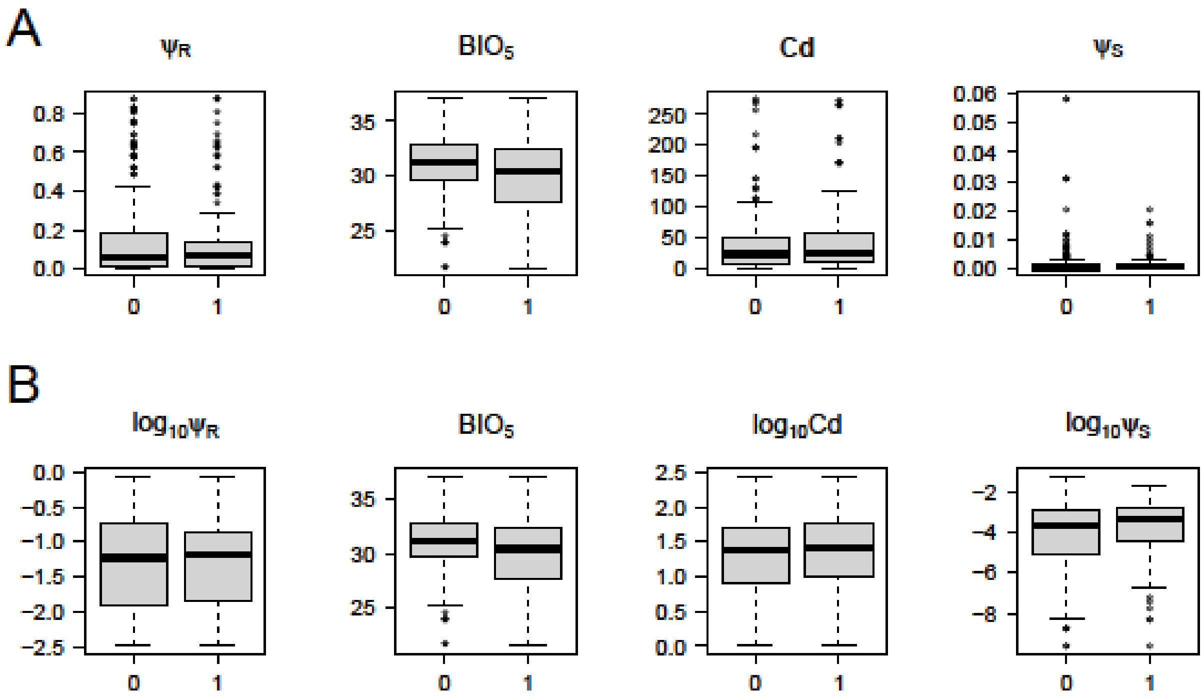

Predictors of the infection risk model were checked for the presence of outliers potentially influencing model parameters estimates. Each predictor was individually checked for the uninfected (0) and infected (1) animals through boxplot visualization **(A)**. Outliers were defined as those values lying outside 1.5 times the interquartile range above the upper quartile and below the lower quartile. After inspection, ψ_R, cattle density (Cd) and ψ_S were transformed on the $\log_{10}$ scale to reduce the potential leverage effect caused by the skewness of their distributions **(B)**. Independent Mann-Whitney-Wilcoxon tests were performed to investigate significant differences between groups (H_0: $\mu_0 = \mu_1$, α=0.05). A significant difference between the means of infected and uninfected animals for BIO_5 (*p-value*=5.203E−05) and $\log_{10}(\psi_S)$ (*p-value*=0.0234) was identified.

2.1.4 Supplementary Figure 4. Additional ADMIXTURE analysis: results.

This figure can be found in the Dryad Digital Repository (doi:10.5061/dryad.sf5j2bf); it depicts the results of the additional ADMIXTURE analysis described in Supplementary Text 3.

2.1.5 Supplementary Figure 5. Correlations between bioclimatic variables and principal components.

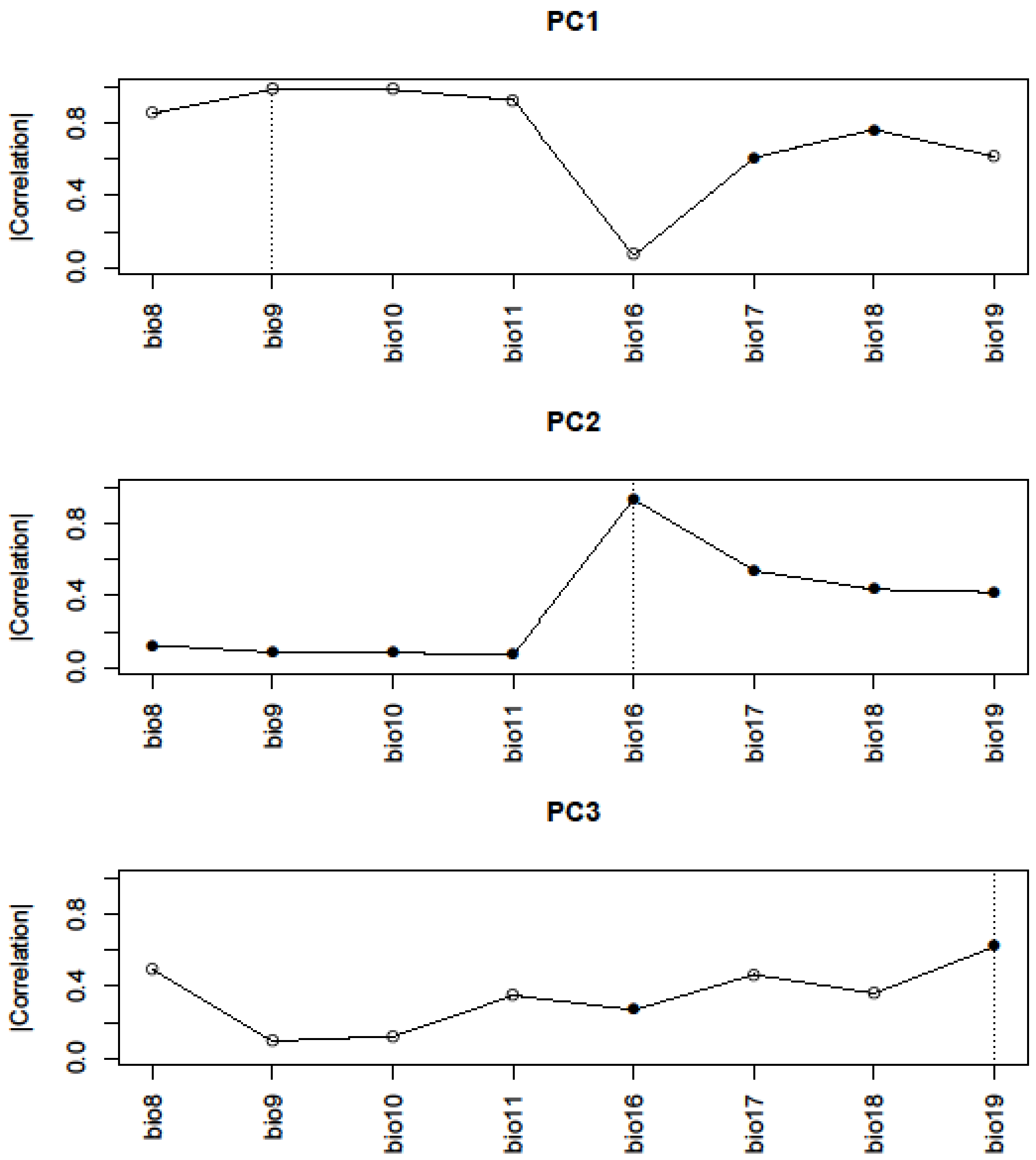

Absolute Pearson's product-moment correlation coefficients between first (**upper plot**), second (**in-between plot**) and third (**lower plot**) principal components and the original bioclimatic variables used to perform PCA. Filled and empty circles indicate positive and negative correlations, respectively. In order to ease interpretation of principal components, a vertical dashed line is positioned in correspondence of bioclimatic variable showing the highest correlation.

2.1.6 Supplementary Figure 6. Candidate *Rhipicephalus appendiculatus* distribution models and model selection.

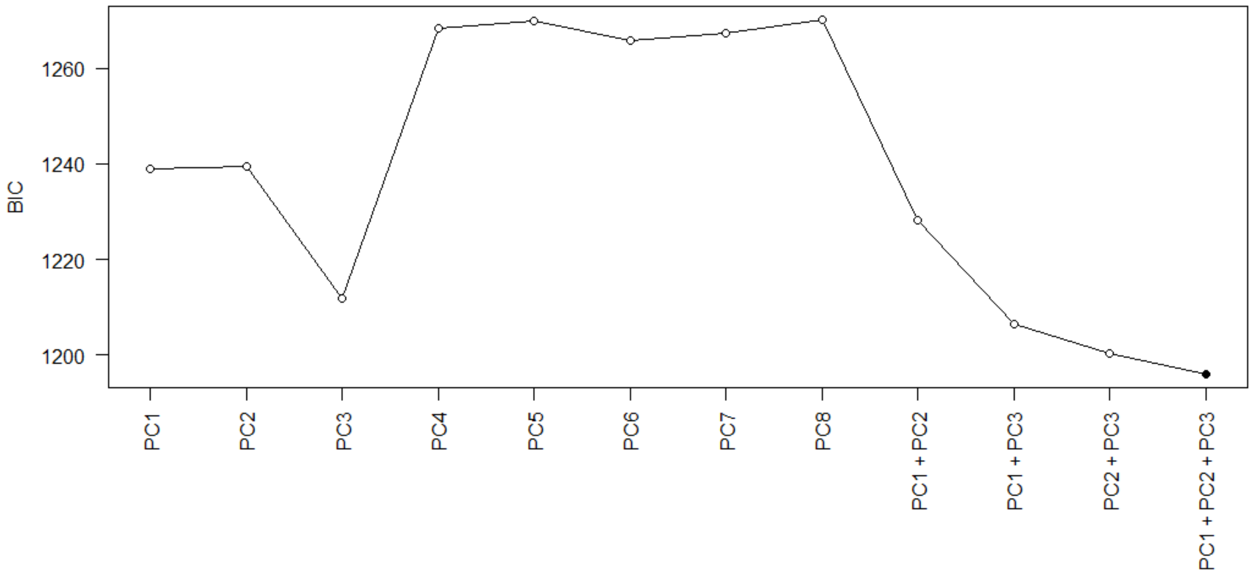

Twelve distribution models (*X*-axis) were tested to describe *R. appendiculatus* distribution in Uganda, and their performances evaluated based on the Bayesian information Criterion (BIC; *Y*-axis). The model including first (PC_1), second (PC_2) and third (PC_3) principal components showed the lowest BIC value (filled circle in the plot), and was therefore retained to predict ψ_R over Uganda.

2.1.7 Supplementary Figure 7. Selected *Rhipicephalus appendiculatus* distribution model with confidence intervals.

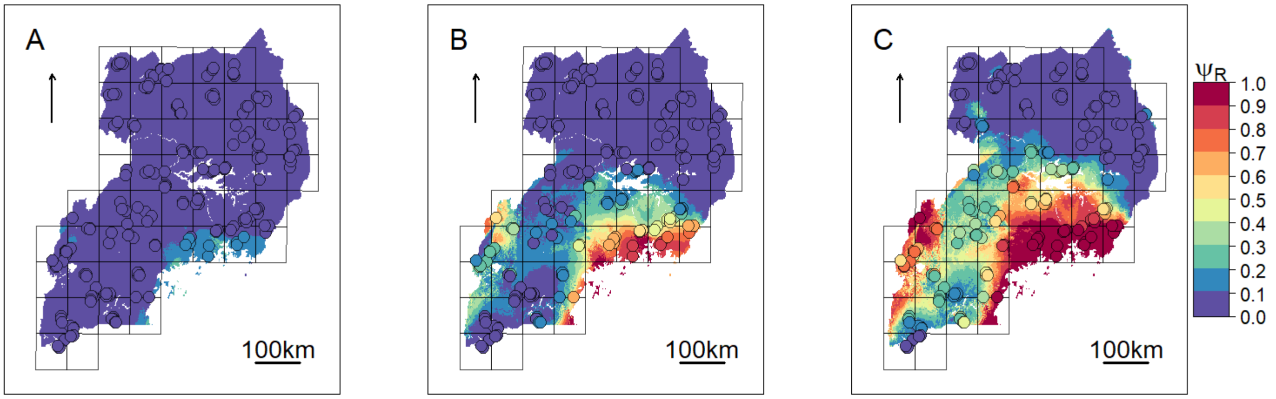

R. appendiculatus occurrence probability (ψ_R) as derived from the selected distribution model **(B)** (see Supplementary Figure 6). Lower **(A)** and upper **(C)** bounds of the 95% confidence intervals around predicted ψ_R are reported. Colour key corresponds to predicted ψ_R, with increasing values from blue to red tones. Sampled farms are represented as circles, and coloured according to the ψ_R value estimated at their geographical location.

2.1.8 Supplementary Figure 8. Candidate *Syncerus caffer* distribution models and model selection.

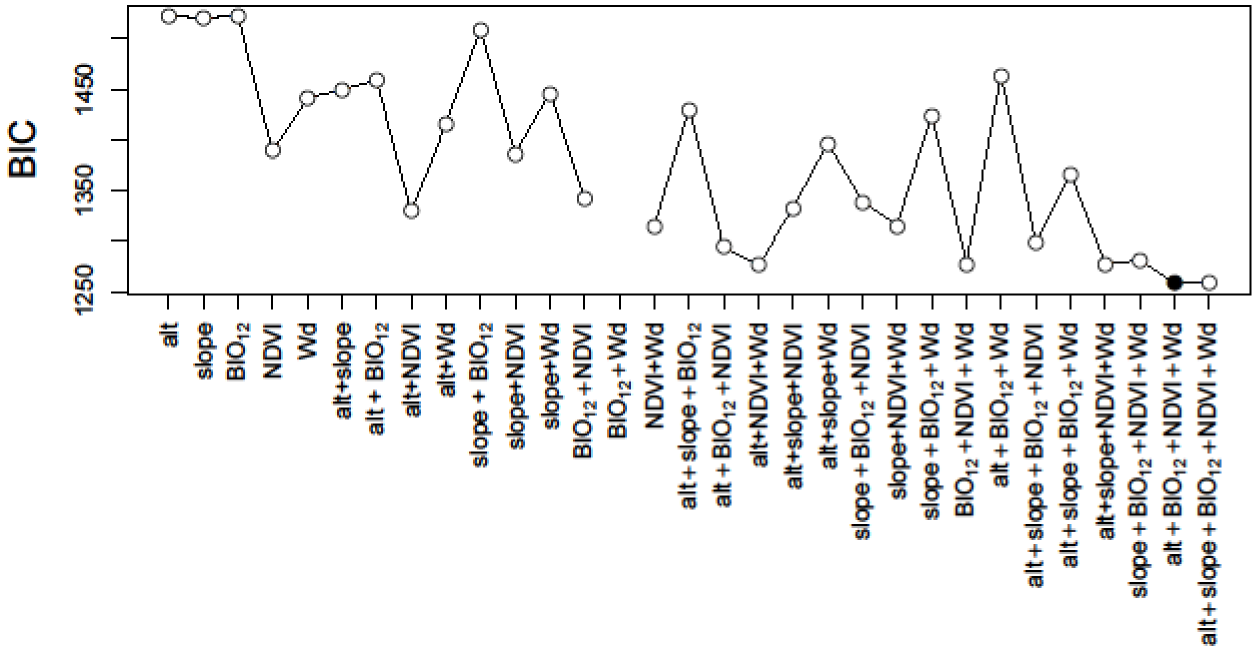

Thirty-one distribution models (*X*-axis) were tested to describe *S. caffer* distribution, and their performances evaluated based on the Bayesian information Criterion (BIC; *Y*-axis). The model including altitude (alt), annual precipitation (BIO_{12}), NDVI, and distance from water (Wd) showed the lowest BIC value (filled circle in the plot), and was therefore retained to represent ψ_S. Model including BIO_{12}+Wd failed to converge, and therefore it does not present any associated BIC.

2.1.9 Supplementary Figure 9. Selected *Syncerus caffer* distribution model with confidence intervals.

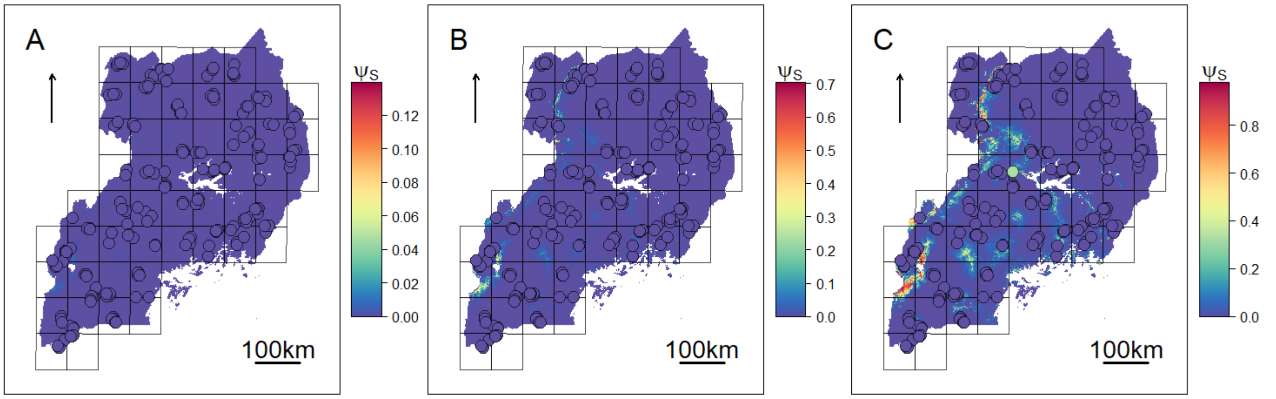

S. caffer occurrence probability (ψ_S) as derived from the selected distribution model **(B)** (see Supplementary Figure 8). Lower **(A)** and upper **(C)** bounds of the 95% confidence intervals around predicted ψ_S are reported. Colour key corresponds to predicted ψ_S, with increasing values from blue to red tones. Sampled farms are represented as circles, and coloured according to the ψ_S value estimated at their geographical location.

2.1.10 Supplementary Figure 10. Map of the Ugandan ancestry components (*K*=4) as derived by ADMIXTURE analysis.

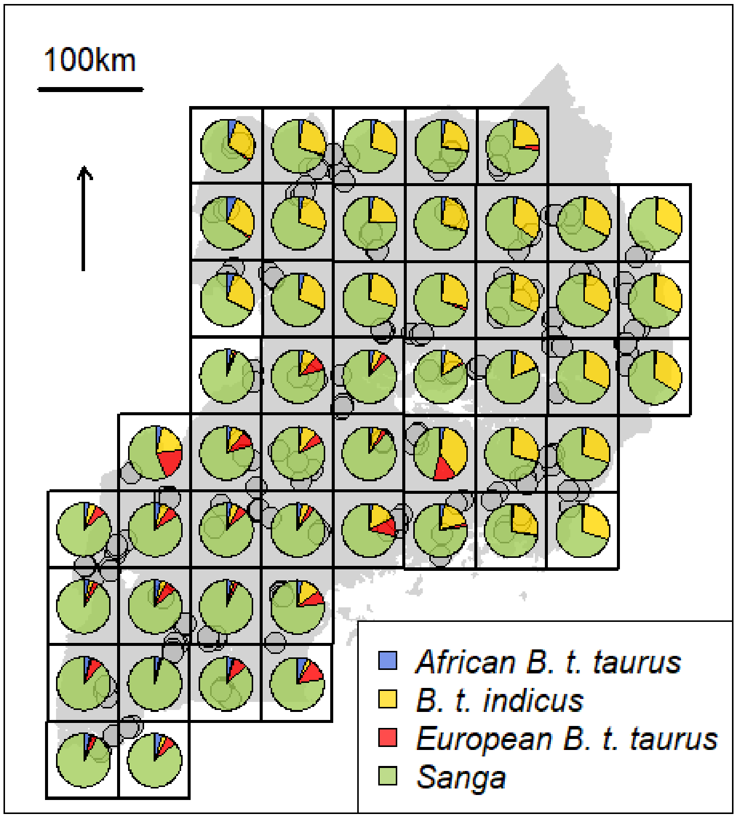

Global ancestry proportions for each cell at *K*=4, the cluster solution used to correct SAMβADA models (see Materials and Methods, main text). Each colour corresponds to a different ancestral gene pool, as inferred by ADMIXTURE analysis (i.e. African *B. t. taurus*, *B. t. indicus*, European *B. t. taurus* and sanga). Sanga and zebuine components appear to have a spatial structure, the sanga component (in green) being more present in south-western Uganda, and the zebuine component (in yellow) in the North-East. Sampled farms are represented as grey circles in the background.

2.1.11 Supplementary Figure 11. Quantile-Quantile plots of the genotype-environment association studies (*K*=4 correction).

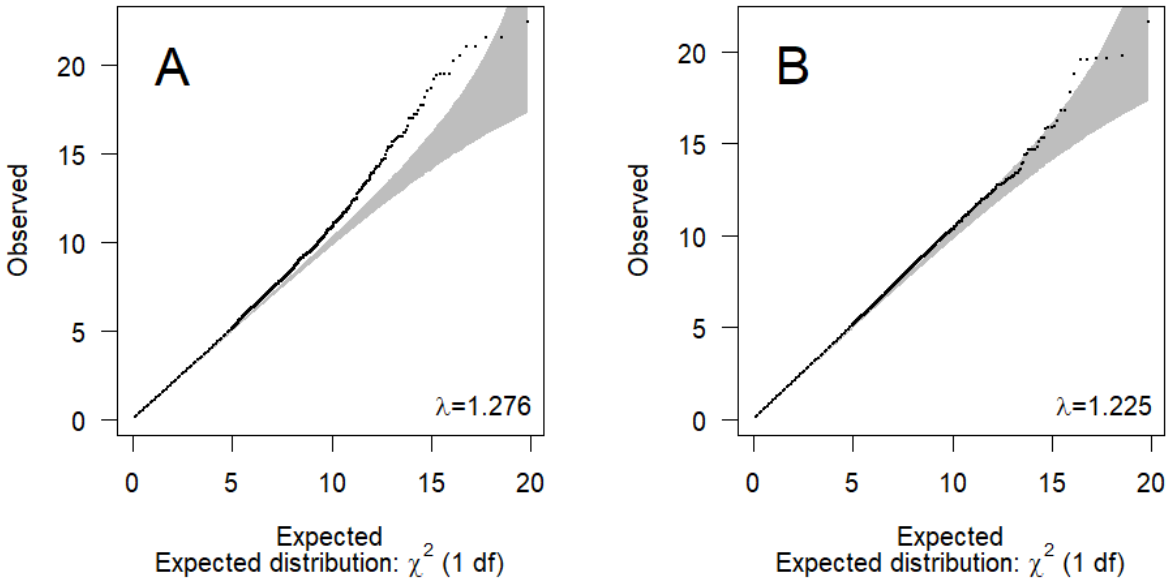

Quantile-Quantile plots of the genotype-environment association studies regarding ψ_R **(A)** and γ **(B)**. Each point is relative to a single likelihood ratio test. In particular, *X*-axis reports the sorted *D*-statistics as derived from a χ^2 distribution with one degree of freedom (i.e. the quantiles of the expected distribution of *D* values); *Y*-axis reports the quantiles of the observed distribution of *D* values. Observed *D* values from both the genotype-environment association studies were divided for their respective genomic inflation factor (λ) prior plotting, in order to correct for overdispersion possibly due to unconsidered sources of bias. Genomic inflation factors (as reported in the bottom right corner of each plot) and Quantile-Quantile plots were calculated and produced with the function `qq.chisq` as embedded in the `snpStats` R package (Clayton, 2015). Overall, observed *D*-statistics from the ψ_R study suggest a higher divergence from the expectation then *D*-statistics from γ association study.

2.1.12 Supplementary Figure 12. Additional SAMβADA analysis (*K*=3 and *K*=16 corrections): results.

Genotype-*R. appendiculatus* occurrence probability association study (*K*=3 correction)

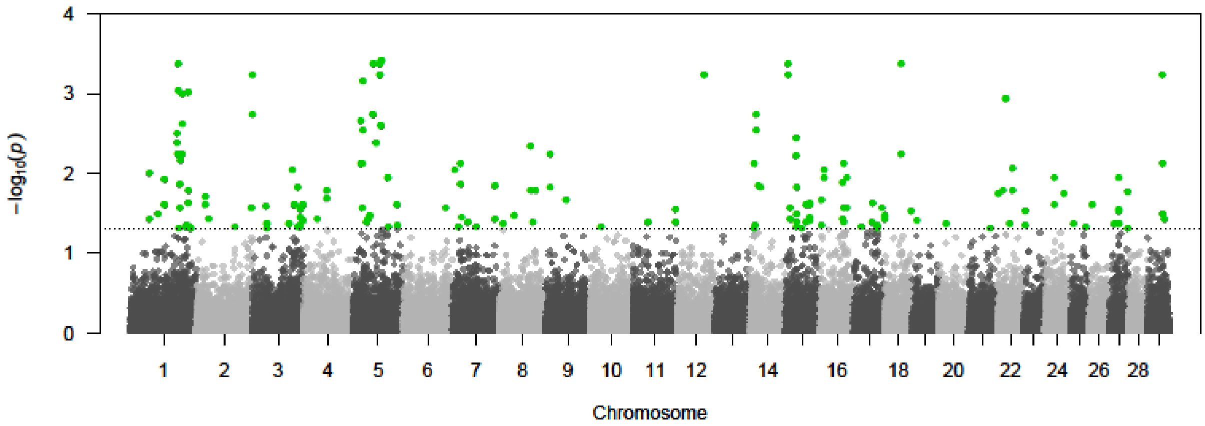

Manhattan plot of the genotype-*R. appendiculatus* occurrence probability (ψ_R) association study for the *K*=3 correction (refer to Supplementary Text 3 for an explanation). *X*-axis reports SNPs chromosomal positions on *B. taurus* chromosomes. On the *Y*-axis, the test statistic *p-values* (*p*) of the associations with ψ_R are shown for each genotype, after the Benjamini-Hochberg (BH) correction, and on the –log10 scale. Nominal significance threshold (α_{BH}=0.05) is displayed as a black dashed line, and significant *p-values* are represented in green. One hundred and twenty-nine SNPs resulted significantly associated with ψ_R. Thirty-eight of them resulted significantly associated also when the *K*=4 correction was used. In particular, Locus BTA-113604 (with genotype AA) resulted from both *K*=3 and *K*=4 analyses, by confirming the association found with the *K*=4 correction (see Table 5A, main text).

Genotype-*R. appendiculatus* occurrence probability association study (*K*=16 correction)

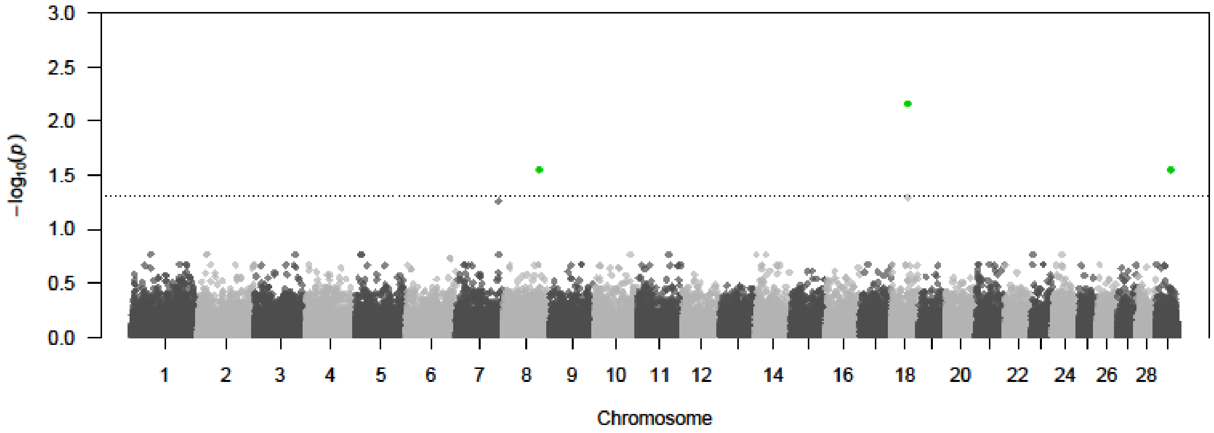

Manhattan plot of the genotype-*R. appendiculatus* occurrence probability (ψ_R) association study for the *K*=16 correction (refer to Supplementary Text 3 for an explanation). *X*-axis reports SNPs chromosomal positions on *B. taurus* chromosomes. On the *Y*-axis, the test statistic *p-values* (*p*) of the associations with ψ_R are shown for each genotype, after the Benjamini-Hochberg (BH) correction,

and on the –log10 scale. Nominal significance threshold (α_{BH}=0.05) is displayed as a black dashed line, and significant *p-values* are represented in green. Three SNPs resulted significantly associated; one of these (ARS-BFGL-NGS-18933, genotype AA, chromosome 29) was present also when the *K*=4 correction was applied, being in linkage with *OPCML* (see Table 5A, main text). The other associated markers (ARS-BFGL-NGS-103237 and ARS-BFGL-BAC-6188, both with genotype AA) were not present in the set of SNPs resulting from the *K*=4 correction.

Genotype-*T. p. parva* infection risk association study (*K*=3 correction)

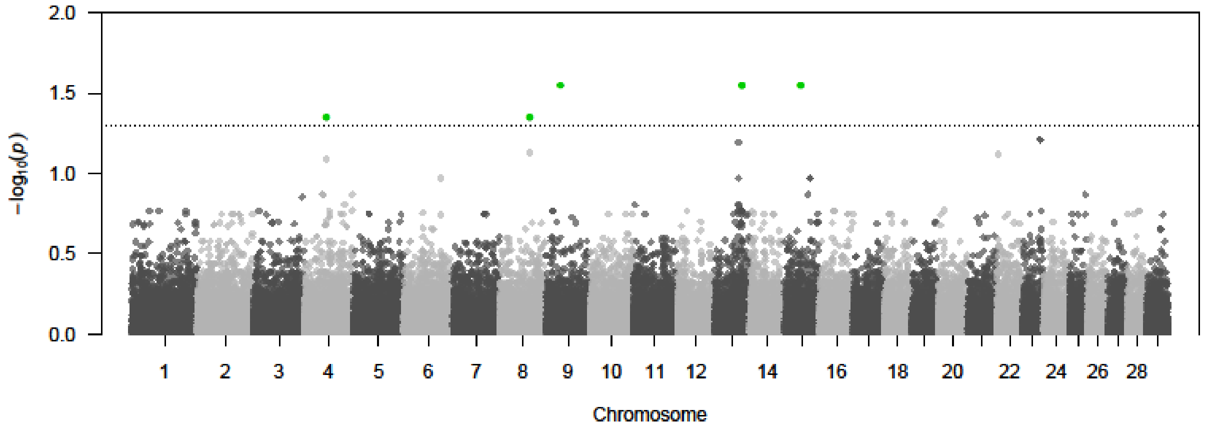

Manhattan plot of the genotype-*T. p. parva* infection risk (γ) association study for the *K*=3 correction (refer to Supplementary Text 3 for an explanation). *X*-axis reports SNPs chromosomal positions on *B. taurus* chromosomes. On the *Y*-axis, the test statistic *p-values* (*p*) of the associations with γ are shown for each genotype, after the Benjamini-Hochberg (BH) correction, and on the –log10 scale. Nominal significance threshold (α_{BH}=0.05) is displayed as a black dashed line, and significant *p-values* are represented in green. Eight SNPs resulted significantly associated with γ; seven of them resulted significantly associated also when the *K*=4 correction was used (see Table 5B, main text), by confirming findings obtained with the *K*=4 correction.

Genotype-*T. p. parva* infection risk association study (*K*=16 correction)

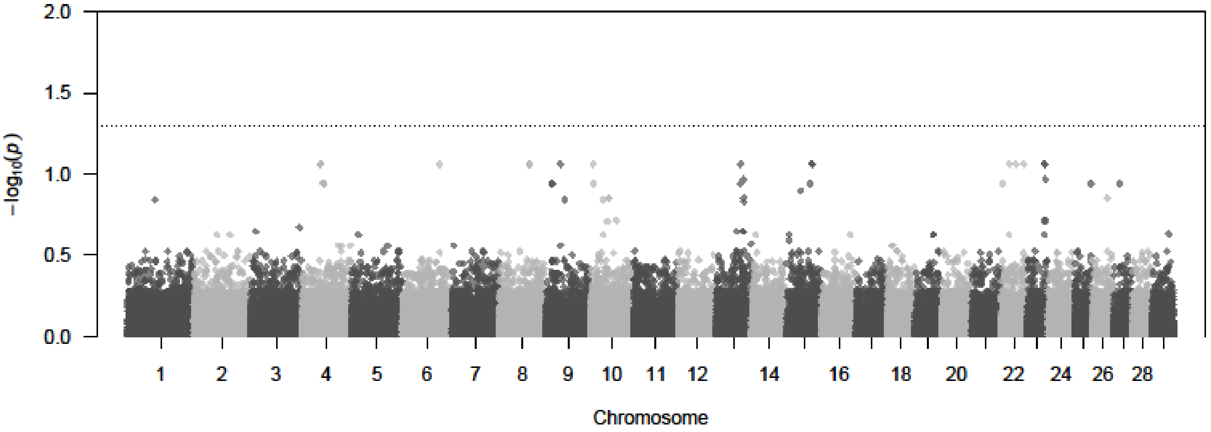

Manhattan plot of the genotype-*T. p. parva* infection risk (γ) association study for the *K*=16 correction (refer to Supplementary Text 3 for an explanation). *X*-axis reports SNPs chromosomal positions on *B. taurus* chromosomes. On the *Y*-axis, the test statistic *p-values* (*p*) of the associations with γ are shown for each genotype, after the Benjamini-Hochberg (BH) correction, and on the –

log10 scale. Nominal significance threshold (α_{BH}=0.05) is displayed as a black dashed line. No marker resulted significantly associated with the *K*=16 correction.

2.1.13 Supplementary Figure 13. *PRKG1* and *SLA2* genomic regions.

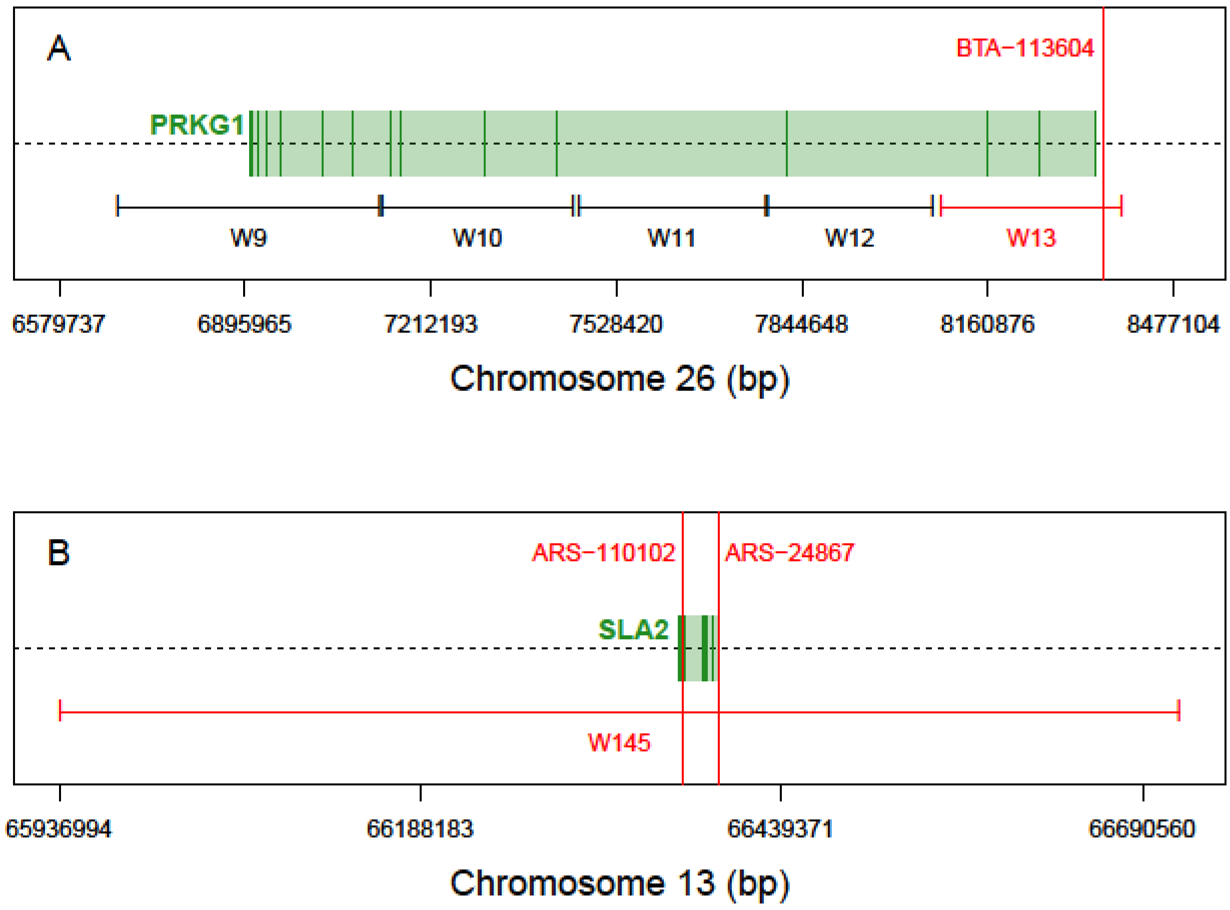

PRKG1 and *SLA2* genomic regions on chromosome 26 **(A)** and 13 **(B)**. Physical positions are reported in base pairs on the *X*-axis. Genes are represented as green rectangles with exons highlighted in dark green. Genomic windows (W) as derived from PCADMIX analysis are also shown in the lower half of each plot. SNPs in linkage disequilibrium with the candidate genes are highlighted by vertical red lines representing their physical position; particularly, BTA-113604 falls within window 13 (position: 8,081,039-8,388,710 bps) on chromosome 26, while ARS-110102 and ARS-24867 within window 145 (positions: 65,937,994-66,715,966 bps) on chromosome 13. The gene name is also shown in red.

2.1.14 Supplementary Figure 14. Spatial representation of PCAdmix assignments (Tharparkar/Muturu comparison).

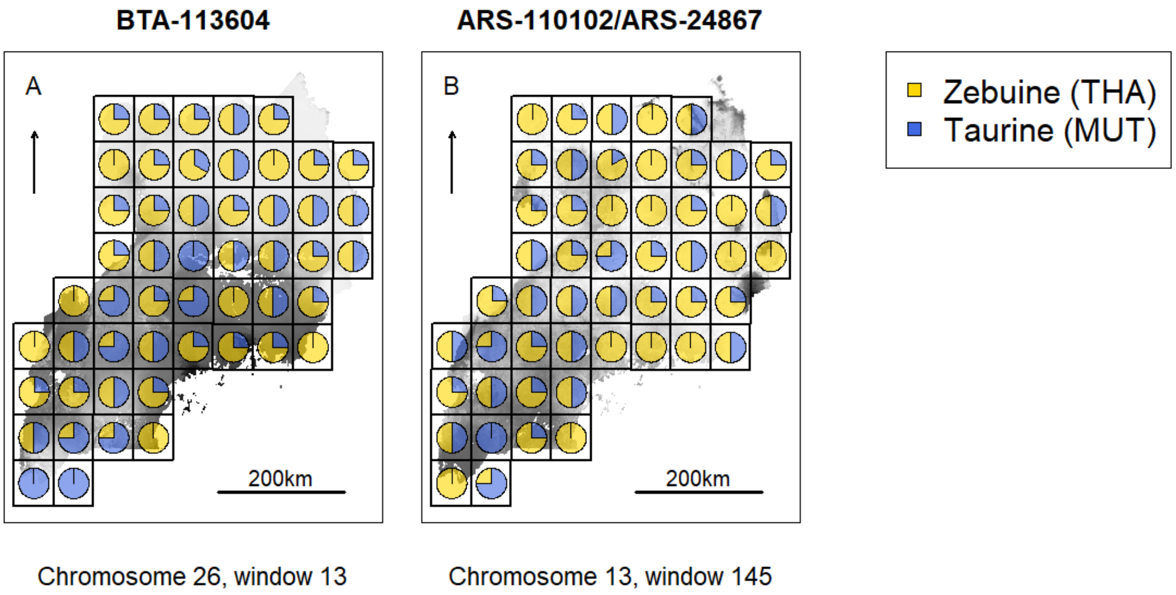

PCAdmix results obtained using Tharparkar (THA) and Muturu (MUT) as references. Pie charts show zebuine (i.e. *B. t. indicus*; THA) and African taurine (i.e. *B. t. taurus*; MUT) proportions per sampling cell (in yellow and blue, respectively) for window 13 on chromosome 26 **(A)**, and windows 145 on chromosome 13 **(B)**. These genomic windows host the SNPs in linkage disequilibrium with *PRKG1* (i.e. BTA-113604) and *SLA2* (i.e. ARS-110102 and ARS-24867). *R. appendiculatus* occurrence probability **(A)** and *T. p. parva* infection risk **(B)** are represented on the background.

2.2 Supplementary Tables

2.2.1 Supplementary Table 1. Composition of the population structure dataset (PSD).

**Breed name**	**Type**	**N**	**Origin**	**Source**
Holstein	European *B. t. taurus*	50	Europe	Decker et al. (2009, 2014); McTavish et al. (2013); The Bovine HapMap Consortium et al. (2009)
Jersey	European *B. t. taurus*	31	Europe	Decker et al. (2009, 2014); McTavish et al. (2013); The Bovine HapMap Consortium et al. (2009)
Hereford	European *B. t. taurus*	50	Europe	Decker et al., (2009, 2014); Gautier et al., (2010); McTavish et al. (2013); The Bovine HapMap Consortium et al. (2009)
Baoule	African *B. t. taurus*	29	Africa (Burkina Faso)	Decker et al. (2014); Gautier et al. (2009)
Lagune	African *B. t. taurus*	30	Africa (Benin)	Decker et al. (2014); Gautier et al. (2009)
N'Dama	African *B. t. taurus*	56	Africa (Ivory Coast. Burkina Faso)	Decker et al. (2014); Gautier et al. (2009, 2010)
Somba	African *B. t. taurus*	30	Africa (Togo)	Decker et al. (2014); Gautier et al. (2009)
Muturu	African *B. t. taurus*	13	Africa (Nigeria)	S[a]
**-**	**Sanga**	**743**	**Africa (Uganda)**	**NEXTGEN project**
Zebu Bororo	Sanga	23	Africa (Chad)	Decker et al. (2014); Gautier et al. (2010)
Zebu Fulani	Sanga	30	Africa (Benin)	Decker et al. (2014); Gautier et al. (2009)
Boran	Sanga	44	Africa (Ethiopia)	Decker et al. (2014); McTavish et al. (2013)
Red Bororo	Sanga	4	Africa (Nigeria)	S

Sokoto Gudali	Sanga	6	Africa (Nigeria)	S
Nganda	Sanga	19	Africa (Uganda)	SH[b]
Sahiwal	Sanga	21	Africa (Kenya/Uganda)	SH
Serere/Teso Zebu	Sanga	15	Africa(Uganda)	SH
Yakanaji	Sanga	13	Africa (Nigeria)	S
Bunaji	Sanga	4	Africa (Nigeria)	S
Karakioja	Sanga	16	Africa(Uganda)	SH
Sahiwal	*B. t. indicus*	17	Asia (Pakistan)	Decker et al. (2009, 2014); McTavish et al. (2013)
Gir	*B. t. indicus*	26	Asia (India)	Decker et al. (2009, 2014); Gautier et al. (2010); McTavish et al. (2013); The Bovine HapMap Consortium et al. (2009)
Tharparkar	*B. t. indicus*	25	Asia (Pakistan)	Decker et al. (2014); S
Kankraj	*B. t. indicus*	10	Asia (India)	Decker et al. (2014)
Nelore	*B. t. indicus*	50	South America (Brazil)	Decker et al.(2009, 2014); Gautier et al., (2010); McTavish et al. (2013); The Bovine HapMap Consortium et al. (2009)

Table reports breeds' names (Breed name), *B. taurus* subspecies (Type), sample size (N), geographical origin (Origin), and data source (Source). Ugandan population from the NEXTGEN project is reported in bold. [a]Genotypes provided by T. Sonstegard; [b]Genotypes provided by T. Sonstegard and H. J. Huson.

2.2.2 Supplementary Table 2. SAMβADA analysis (K=4 correction): significant associations with *Rhipicephalus appendiculatus* occurrence probability after correction for multiple testing.

SNP ID (genotype)	Chr	Position	D	p_{BH}	β_0	ψ_R	PC_1	PC_2	PC_3
ARS-BFGL-NGS-18933 (GG)	29	34650967	28.607	0.005	-9.272	10.365	-1.401	-0.705	-1.344
Hapmap51626-BTA-73514 (AA)	5	48834486	27.442	0.005	6.036	-6.006	0.085	0.804	-0.996
Hapmap51626-BTA-73514 (AG)	5	48834486	27.442	0.005	-6.036	6.006	-0.085	-0.804	0.996
Hapmap51479-BTA-66720 (GG)	5	64330943	26.833	0.005	4.877	-4.676	0.106	0.864	-0.941
Hapmap55537-rs29016129 (GG)	5	64380551	26.833	0.005	4.877	-4.676	0.106	0.864	-0.941
BTA-46975-no-rs (GG)	5	68220538	26.173	0.006	5.986	-5.557	0.123	0.761	-1.57
BTA-46975-no-rs (CG)	5	68220538	25.759	0.007	-6.226	5.793	-0.221	-0.719	1.657
ARS-BFGL-NGS-11580 (CC)	1	114981065	24.841	0.007	4.508	-4.133	0.04	0.78	-1.166
Hapmap51479-BTA-66720 (AG)	5	64330943	24.812	0.007	-4.953	4.641	-0.178	-0.763	0.966
Hapmap55537-rs29016129 (AG)	5	64380551	24.812	0.007	-4.953	4.641	-0.178	-0.763	0.966
ARS-BFGL-BAC-6188 (AA)	18	38850678	24.747	0.007	1.214	-2.049	0.439	-0.242	-0.185
Hapmap50589-BTA-119599 (AG)	15	7989843	24.477	0.007	-5.226	4.821	-0.226	-0.617	1.279
Hapmap36616-SCAFFOLD310212_1822 (AA)	5	23171537	23.856	0.01	5.786	-5.456	-0.283	0.611	-0.936
UA-IFASA-6140 (AA)	7	102472846	23.646	0.01	2.733	6.062	0.525	-0.028	-0.434
Hapmap50589-BTA-119599 (GG)	15	7989843	23.15	0.012	5.089	-4.606	0.177	0.66	-1.192
BTB-00839408 (AG)	22	18978658	22.586	0.014	-5.983	5.685	-0.336	-0.611	1.113
BTB-00839408 (AA)	22	18978658	22.586	0.014	5.983	-5.685	0.336	0.611	-1.113
UA-IFASA-5221 (GG)	5	18739471	22.267	0.016	4.807	-4.386	0.354	0.745	-0.85
Hapmap34056-BES2_Contig421_810 (AG)	1	138178130	21.966	0.017	-5.527	4.947	0.21	-0.618	1.091
Hapmap34056-BES2_Contig421_810 (GG)	1	138178130	21.966	0.017	5.527	-4.947	-0.21	0.618	-1.091
ARS-BFGL-NGS-11580 (AC)	1	114981065	21.685	0.017	-4.559	3.954	-0.009	-0.757	1.205
Hapmap57868-rs29020458 (AA)	24	22746291	21.675	0.017	-1.163	1.855	0.362	-0.089	-0.068
BTA-97369-no-rs (GG)	14	25887784	21.641	0.017	-0.459	-1.964	0.071	-0.116	0.252
BTB-00292673 (AA)	7	4953801	21.101	0.022	-69.144	86.381	5.517	-8.483	4.851
ARS-BFGL-BAC-6188 (CC)	18	38850678	20.782	0.024	-4.248	3.811	-0.445	0.796	-0.287
BTB-01283856 (AG)	12	65131442	20.666	0.024	-5.301	4.578	-0.001	-0.099	1.392
BTB-01283856 (GG)	12	65131442	20.666	0.024	5.301	-4.578	0.001	0.099	-1.392
BTB-01058465 (GG)	1	113745976	20.318	0.025	3.954	-3.511	0.297	0.859	-0.806
BTB-01058465 (AG)	1	113745976	20.318	0.025	-3.954	3.511	-0.297	-0.859	0.806

ARS-BFGL-NGS-37845 (AG)	5	48633731	20.308	0.025	-6.562	5.87	-0.594	-0.713	1.572
ARS-BFGL-NGS-37845 (AA)	5	48633731	20.308	0.025	6.562	-5.87	0.594	0.713	-1.572
ARS-BFGL-NGS-103237 (AA)	8	87067969	20.231	0.026	-9.864	-48.49	-0.672	-0.203	0.69
ARS-BFGL-NGS-37889 (AA)	9	10370879	20.168	0.026	4.558	-4.002	0.293	0.762	-0.821
BTB-01109852 (AG)	14	15585398	20.056	0.026	-4.562	3.955	-0.256	-0.425	1.102
ARS-BFGL-NGS-32909 (CC)	5	67846632	19.931	0.027	4.744	-4.092	0.142	0.637	-1.042
ARS-BFGL-NGS-32909 (AC)	5	67846632	19.931	0.027	-4.744	4.092	-0.142	-0.637	1.042
UA-IFASA-6140 (AG)	7	102472846	19.716	0.029	-2.708	-5.334	-0.492	0.047	0.373
Hapmap36616-SCAFFOLD310212_1822 (AG)	5	23171537	19.639	0.029	-5.793	5.087	0.331	-0.57	1.031
Hapmap50904-BTA-17187 (AA)	1	124692274	19.632	0.029	2.619	-2.313	-0.255	-0.074	-0.531
ARS-BFGL-NGS-402 (GG)	29	35698376	19.561	0.029	2.169	-2.178	-0.14	0.476	-0.448
ARS-BFGL-NGS-110339 (AA)	1	111495891	19.521	0.029	3.467	-3.013	0.029	0.676	-0.91
ARS-BFGL-NGS-11845 (AA)	27	21512601	19.276	0.032	5.262	-4.664	0.309	0.848	-0.671
ARS-BFGL-NGS-16947 (AA)	15	26629340	19.053	0.035	4.868	-3.985	-0.298	0.815	-1.086
Hapmap39895-BTA-15668 (CC)	5	13311842	18.725	0.039	5.563	-5.094	-0.131	1.103	0.437
Hapmap39895-BTA-15668 (AC)	5	13311842	18.725	0.039	-5.563	5.094	0.131	-1.103	-0.437
ARS-BFGL-NGS-110339 (AC)	1	111495891	18.669	0.039	-3.509	2.979	-0.004	-0.678	0.938
BTB-01956180 (AG)	27	43656445	18.664	0.039	-0.987	1.695	-0.024	-0.098	0.052
UA-IFASA-5221 (AG)	5	18739471	18.645	0.039	-4.779	4.093	-0.336	-0.723	0.883
ARS-BFGL-NGS-99064 (AA)	1	44813737	18.587	0.039	0.351	1.763	-0.395	0.087	-0.013
ARS-BFGL-NGS-63882 (GG)	2	135994305	18.376	0.043	6.945	-5.619	0.37	0.159	-2.585
Hapmap34409-BES7_Contig244_858 (AA)	1	120149924	18.213	0.044	4.258	-3.515	-0.082	0.758	-0.907
Hapmap39826-BTA-37247 (CC)	15	12975036	18.186	0.044	3.195	-2.786	-0.253	0.356	0.025
ARS-BFGL-NGS-39898 (GG)	22	1319636	18.165	0.044	-0.475	-1.802	0.035	-0.127	0.116
Hapmap39826-BTA-37247 (AC)	15	12975036	18.157	0.044	-3.232	2.795	0.251	-0.334	0.073
ARS-BFGL-NGS-16947 (AC)	15	26629340	18.155	0.044	-4.84	3.928	0.295	-0.786	0.991
Hapmap50904-BTA-17187 (AG)	1	124692274	18.126	0.044	-2.614	2.241	0.255	0.078	0.483
BTA-113604-no-rs (AA)	26	8356096	18.024	0.046	-7.089	6.883	-1.157	-0.404	0.252
BTA-60607-no-rs (AA)	25	6742260	17.967	0.046	-0.823	-1.996	-0.207	0.045	0.123
Hapmap31116-BTA-143121 (AA)	8	75973285	17.854	0.048	2.105	-1.974	-0.362	-0.215	-0.157
ARS-BFGL-NGS-104610 (AG)	11	104293559	17.742	0.049	-0.272	-1.7	-0.127	0.078	0.192

Hapmap51155-BTA-11643 (AA)	24	38086180	17.721	0.049	-364.868	501.77	24.71	-32.84	27.863
ARS-BFGL-NGS-37889 (AT)	9	10370879	17.695	0.049	-4.551	3.821	-0.281	-0.767	0.788
ARS-BFGL-BAC-31319 (AA)	23	4847028	17.683	0.049	-0.973	-2.1	-0.238	-0.131	0.184

For each SNP (and genotype), the chromosome (Chr), physical position in base pairs (Position), the Likelihood Ratio test statistic (*D*), and the *p-value* associated with *D* after multiple testing correction (p_{BH}) are shown. Regression coefficients of the alternative models as estimated by SAMβADA are also reported, i.e. the model intercept (β_0), the coefficient associated with tick occurrence probability (ψ_R), and the coefficients associated with the population structure predictors (PC1, PC2 and PC3, respectively). Regression coefficients are expressed on the logit scale, and associations are sorted for decreasing values of the *D*-statistics.

2.2.3 **Supplementary Table 3.** SAMβADA analysis (*K*=4 correction): significant associations with *Theileria parva parva* infection risk after correction for multiple testing.

**SNP ID (genotype)**	**Chr**	**Position**	***D***	p_{BH}	β_0	γ	PC_1	PC_2	PC_3
ARS-BFGL-NGS-112656 (AA)	13	66336246	26.507	0.019	1.799	-7.131	-0.295	0.282	-0.032
ARS-BFGL-NGS-110102 (GG)	13	66370867	24.254	0.019	1.76	-6.748	-0.263	0.286	-0.018
BTA-33234-no-rs (GG)	13	66291997	24.06	0.019	1.889	-6.881	-0.239	0.286	-0.044
ARS-BFGL-NGS-24867 (AA)	13	66395465	24.045	0.019	1.824	-6.785	-0.236	0.244	-0.121
Hapmap39482-BTA-36746 (CC)	15	40279014	24.01	0.019	5.452	-17.615	-1.162	0.495	-0.622
Hapmap39482-BTA-36746 (AC)	15	40279014	24.01	0.019	-5.452	17.615	1.162	-0.495	0.622
BTB-00384802 (AA)	9	34050782	23.05	0.027	-0.42	-6.085	0.05	-0.074	0.056
BTB-01298953 (AA)	4	54930726	21.786	0.045	1.243	6.926	0.166	0.171	-0.249

For each SNP (and genotype), the chromosome (Chr), physical position in base pairs (Position), the Likelihood Ratio test statistic (*D*), and the *p-value* associated with *D* after multiple testing correction (p_{BH}) are shown. Regression coefficients of the alternative models as estimated by SAMβADA are also reported, i.e. the model intercept (β_0), the coefficient associated with *T. p. parva* infection risk (γ), and the coefficients associated with the population structure predictors (PC1, PC2 and PC3, respectively). Regression coefficients are expressed on the logit scale, and associations are sorted for decreasing values of the *D*-statistics.

3 References

Barbato, M., Hailer, F., Orozco-terWengel, P., Kijas, J., Mereu, P., Cabras, P., et al. (2017). Genomic signatures of adaptive introgression from European mouflon into domestic sheep. *Sci. Rep.* 7. doi:10.1038/s41598-017-07382-7.

Budde, M. (2018). [EXTERNAL] FEWS Contact Form | Data & Science Support.

Clayton, D. (2015). *snpStats: SnpMatrix and XSnpMatrix classes and methods. R package version 1.28.0.*

Decker, J. E., McKay, S. D., Rolf, M. M., Kim, J., Molina Alcalá, A., Sonstegard, T. S., et al. (2014). Worldwide Patterns of Ancestry, Divergence, and Admixture in Domesticated Cattle. *PLoS Genet.* 10, e1004254. doi:10.1371/journal.pgen.1004254.

Decker, J. E., Pires, J. C., Conant, G. C., McKay, S. D., Heaton, M. P., Chen, K., et al. (2009). Resolving the evolution of extant and extinct ruminants with high-throughput phylogenomics. *Proc. Natl. Acad. Sci.* 106, 18644–18649.

Gautier, M., Flori, L., Riebler, A., Jaffrézic, F., Laloé, D., Gut, I., et al. (2009). A whole genome Bayesian scan for adaptive genetic divergence in West African cattle. *BMC Genomics* 10, 550. doi:10.1186/1471-2164-10-550.

Gautier, M., Laloë, D., and Moazami-Goudarzi, K. (2010). Insights into the Genetic History of French Cattle from Dense SNP Data on 47 Worldwide Breeds. *PLoS ONE* 5, e13038. doi:10.1371/journal.pone.0013038.

Heneghan, C., Thompson, M., Billingsley, M., and Cohen, D. (2011). Data from: Medical-device recalls in the UK and the device-regulation process: retrospective review of safety notices and alerts. *Dryad Digit. Repos.* doi:https://doi.org/10.5061/dryad.585t4.

Jolliffe, I. (2002). *Principal Component Analysis*. New York: Springer.

Kopelman, N. M., Mayzel, J., Jakobsson, M., Rosenberg, N. A., and Mayrose, I. (2015). Clumpak: a program for identifying clustering modes and packaging population structure inferences across K. *Mol. Ecol. Resour.* 15, 1179–1191. doi:10.1111/1755-0998.12387.

McTavish, E. J., Decker, J. E., Schnabel, R. D., Taylor, J. F., and Hillis, D. M. (2013). New World cattle show ancestry from multiple independent domestication events. *Proc. Natl. Acad. Sci.* 110, E1398–E1406.

Muscarella, R., Galante, P. J., Soley-Guardia, M., Boria, R. A., Kass, J. M., Uriarte, M., et al. (2014). ENMeval: An R package for conducting spatially independent evaluations and estimating optimal model complexity for MAXENT ecological niche models. *Methods Ecol. Evol.* 5, 1198–1205. doi:10.1111/2041-210X.12261.

Royle, J. A., Chandler, R. B., Yackulic, C., and Nichols, J. D. (2012). Likelihood analysis of species occurrence probability from presence-only data for modelling species distributions: *Likelihood analysis of presence-only data*. *Methods Ecol. Evol.* 3, 545–554. doi:10.1111/j.2041-210X.2011.00182.x.

The Bovine HapMap Consortium, Gibbs, R. A., Taylor, J. F., Van Tassell, C. P., Barendse, W., Eversole, K. A., et al. (2009). Genome-Wide Survey of SNP Variation Uncovers the Genetic Structure of Cattle Breeds. *Science* 324, 528–532. doi:10.1126/science.1167936.

US Geological Survey (USGS) Earth Resources Observation and Science (EROS) Center - Famine Early Warning Systems Network (FEWS NET). Available at: https://earlywarning.usgs.gov/fews.
